# Admixture-driven structural variation diversity and its functional implications

**DOI:** 10.1093/nsr/nwaf527

**Published:** 2025-11-21

**Authors:** Haiyi Lou, Yimin Wang, Yu Chai, Zhilin Ning, Ruiqing Fu, Yan Lu, Bo Xie, Sen Ma, Yuwen Pan, Yang Gao, Dongsheng Lu, Xinyue Bai, Yajun Yang, Dolikun Mamatyusupu, Shuhua Xu

**Affiliations:** State Key Laboratory of Genetics and Development of Complex Phenotypes, Center for Evolutionary Biology, School of Life Sciences, Fudan University, Shanghai 200438, China; Shanghai Institute of Nutrition and Health, Shanghai Institutes for Biological Sciences, University of Chinese Academy of Sciences, Chinese Academy of Sciences, Shanghai 200031, China; State Key Laboratory of Genetics and Development of Complex Phenotypes, Center for Evolutionary Biology, School of Life Sciences, Fudan University, Shanghai 200438, China; Shanghai Institute of Nutrition and Health, Shanghai Institutes for Biological Sciences, University of Chinese Academy of Sciences, Chinese Academy of Sciences, Shanghai 200031, China; Shanghai Institute of Nutrition and Health, Shanghai Institutes for Biological Sciences, University of Chinese Academy of Sciences, Chinese Academy of Sciences, Shanghai 200031, China; State Key Laboratory of Genetics and Development of Complex Phenotypes, Center for Evolutionary Biology, School of Life Sciences, Fudan University, Shanghai 200438, China; Ministry of Education Key Laboratory of Contemporary Anthropology, Fudan University, Shanghai 201203, China; Shanghai Institute of Nutrition and Health, Shanghai Institutes for Biological Sciences, University of Chinese Academy of Sciences, Chinese Academy of Sciences, Shanghai 200031, China; Shanghai Institute of Nutrition and Health, Shanghai Institutes for Biological Sciences, University of Chinese Academy of Sciences, Chinese Academy of Sciences, Shanghai 200031, China; Shanghai Institute of Nutrition and Health, Shanghai Institutes for Biological Sciences, University of Chinese Academy of Sciences, Chinese Academy of Sciences, Shanghai 200031, China; State Key Laboratory of Genetics and Development of Complex Phenotypes, Center for Evolutionary Biology, School of Life Sciences, Fudan University, Shanghai 200438, China; State Key Laboratory of Genetics and Development of Complex Phenotypes, Center for Evolutionary Biology, School of Life Sciences, Fudan University, Shanghai 200438, China; Shanghai Institute of Nutrition and Health, Shanghai Institutes for Biological Sciences, University of Chinese Academy of Sciences, Chinese Academy of Sciences, Shanghai 200031, China; School of Life Science and Technology, ShanghaiTech University, Shanghai 201210, China; Ministry of Education Key Laboratory of Contemporary Anthropology, Fudan University, Shanghai 201203, China; College of Life Sciences and Technology, Xinjiang University, Urumqi 830046, China; State Key Laboratory of Genetics and Development of Complex Phenotypes, Center for Evolutionary Biology, School of Life Sciences, Fudan University, Shanghai 200438, China; School of Life Science and Technology, ShanghaiTech University, Shanghai 201210, China

**Keywords:** Uyghur, population admixture, structural variation (SV), non-allelic homologous recombination (NAHR), local adaptation, ancestry bias, evolutionary dynamics

## Abstract

Population admixture is a potent evolutionary force shaping genomic diversity, yet its influence on the dynamics and functional consequences of structural variation (SV) remains poorly understood. Here, we present a comprehensive whole-genome sequencing analysis of SVs in the Uyghurs, a model admixed Eurasian population with distinct Western and Eastern ancestral contributions. We identified 9965 high-confidence SVs, revealing that Uyghurs exhibit 32% novel SVs and 1.19-fold greater SV-transcription diversity compared to their ancestral source populations. Crucially, SV diversity follows a non-linear parabolic relationship with ancestry proportions (r = 0.94), peaking when Western/Eastern ancestry contributions are balanced. Admixture-induced recombination at ancestry junctions creates SV hotspots via non-allelic homologous recombination (NAHR), with 60% of post-admixture SVs flanked by homologous repeats. Ancestry-divergent SVs disproportionately regulate gene expression, while admixed variant combinations (e.g. *HLA-B* and *FOXO6* loci) disrupt immune/metabolic pathways via additive regulatory effects. Strikingly, while Uyghurs harbor elevated SV diversity, the burden of pathogenic variants remains comparable to ancestral populations, suggesting buffering mechanisms against genetic load. Evolutionarily, younger SVs tend to be larger in size, exert stronger regulatory impacts and exhibit higher predicted pathogenicity. These findings establish admixture as a dual force of genomic diversification and functional equilibrium, bridging evolutionary dynamics with biomedical insights. Our work underscores the necessity of SV-aware approaches in genetic medicine and highlights how admixed populations broaden genomic diversity beyond ancestral boundaries through novel variant combinations.

## INTRODUCTION

Structural variation (SV) is defined as a segment of DNA with 50 bp-larger presenting variation, such as deletion, duplication, inversion, insertion and translocation, in the human genome [1]. In human evolution, SVs are a large source of genetic variations in addition to the single-nucleotide variants (SNVs) [2], involved in the divergence between humans and apes [3], and contributing to human genetic polymorphisms [4]. The functional impact of these variants is also considerable. On the molecular function level, SVs could affect gene expression by disrupting genes, changing gene dosage, altering the regulation and interrupting the 3D structure of the chromosome [5,6]. On the phenotypic level, the SVs are associated with adaptive traits [7,8], affecting the disease risks [9] and causing human diseases like neurological diseases [10,11] and cancers [12]. Recently, the detection of SVs has been improved by long-read sequencing technology [e.g. single-molecule real-time (SMRT) sequencing] [13–15], but the high cost hinders large-scale discovery on a population level. Short-read sequencing, or next-generation sequencing (NGS), still plays a major role, and several large projects have applied NGS to detect SVs in diverse populations [4,16–18]. However, the study of admixed populations, especially the Eurasian populations, is underrepresented.

Admixed populations at continental crossroads provide unique opportunities to investigate how recombination between divergent ancestries shapes SV dynamics [19–22]. The Xinjiang Uyghur (XJU) population, the focal population of this study with its well-characterized West–East Eurasian admixture history and balanced ancestral contributions (∼50% each), serves as an ideal model system to examine SV emergence and selection patterns post-admixture [23–26]. Previous work using SNVs identified a two-wave admixture timeline, with the most recent large-scale gene flow occurring approximately 750 years ago [27]. While copy number variation (CNV) studies confirmed this admixture signature, microarray-based approaches were limited to detecting large (>1 kb) deletions/duplications, leaving smaller SVs and complex types like insertions/inversions unexplored [28]. This population’s distinct recombination landscape—featuring abundant ancestry junctions from recent admixing—creates a natural experiment to study how inter-ancestry recombination drives SV formation through mechanisms like non-allelic homologous recombination (NAHR). Furthermore, its balanced ancestry proportions enable systematic analysis of how SV diversity scales with admixture ratios, and how selection acts on admixed haplotypes. These characteristics address critical gaps in understanding SV evolution in recently admixed genomes, particularly regarding: (i) the interplay between recombination hotspots and SV formation; (ii) the regulatory consequences of ancestry-specific SV combinations; and (iii) the evolutionary equilibrium between diversity amplification and pathogenic burden maintenance.

## RESULTS

### Genomic profiles of SVs in the XJU population

**Figure 1.**
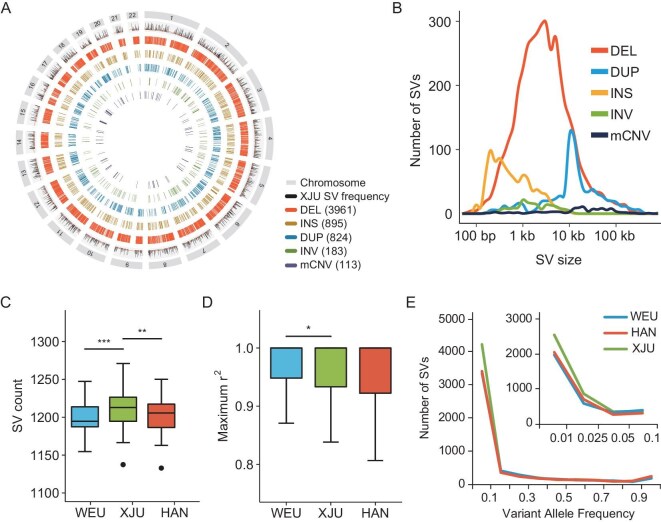
SV profiles in the XJU genomes. (A) Circular plot of 5976 non-redundant SVs in the 85 XJU samples. (B) Size distribution of different SV types. (C) SV count of each genome. (D) LD between SV and flanking SNVs. (E) SV allele frequency distribution for three populations: blue, WEU; red, HAN; green, XJU. The inset graph shows the allele frequencies that were <0.1. DEL, deletion; DUP, duplication; INS, insertion; INV, inversion. *, ** and *** denote the significance levels of 0.05, 0.01 and 0.001, respectively.

We sequenced whole genomes of 92 XJU and 90 Han Chinese (HAN) individuals to high coverage (>30×). The HAN genomes were used as a proxy of East Eurasian ancestral source reference. In addition, we combined the West Eurasian (WEU) samples from Simons Genome Diversity Project (SGDP), which were also sequenced to at least 30× using Illumina technology [4], as a proxy of WEU ancestral source reference. We developed a pipeline with high precision (∼0.87) to detect SVs from the NGS data (Fig. S1; Table S1; Supplementary Materials and Methods). Using this SV discovery pipeline, we obtained a median of 4440 calls for each individual in the XJU population. After further sample-level and variant-level filtration, the high-confidence callset contained a total of 9965 SVs including 7088 deletions, 1567 duplications, 126 multi-allelic CNVs (mCNVs), 901 non-repetitive insertions and 283 inversions from the merged dataset of 85 XJU, 89 HAN and 67 SGDP WEU samples, which were used in the subsequent analysis (Fig. 1A; Table S2). Among these SVs, we found 5976 polymorphic SVs in the 85 XJU samples, and 32.6% of these SVs were novel compared with the Database of Genomic Variants (DGV) [29] under a reciprocal length-overlapping threshold of 50% (Fig. S2). Different SV types presented distinct variant lengths ranging from 57 bp to 767 kb, with a median of 2504 bp (Fig. 1B). On average, each XJU sample carried 1192 variants, which was significantly more than that of HAN and SGDP WEU samples (*P* < 0.05, *t*-test; Fig. 1C), showing increased SVs in the Eurasian admixed individuals. Nearly 83.0% of the XJU SVs with minor allele frequency (MAF) of >0.05 were in strong linkage disequilibrium (LD) with flanking SNVs (r^2^ > 0.8) (Fig. S3). The overall LD pattern between SNVs and SVs in the XJU population is intermediate between the two reference populations. Specifically, the SNV–SV LD in XJU samples is significantly weaker compared with the SGDP WEU samples (*P* < 0.05, Wilcoxon test; Fig. 1D) but slightly stronger (though not statistically significant) than the HAN samples. When comparing variant allele frequency (VAF) of XJU to the West and East Eurasian reference populations, the two reference populations show a similar distribution, but the XJU population has an excessive number of SVs of low and moderate frequency (VAF < 0.1; Fig. 1E; Fig. S4).

### Genetic admixture of SVs

**Figure 2.**
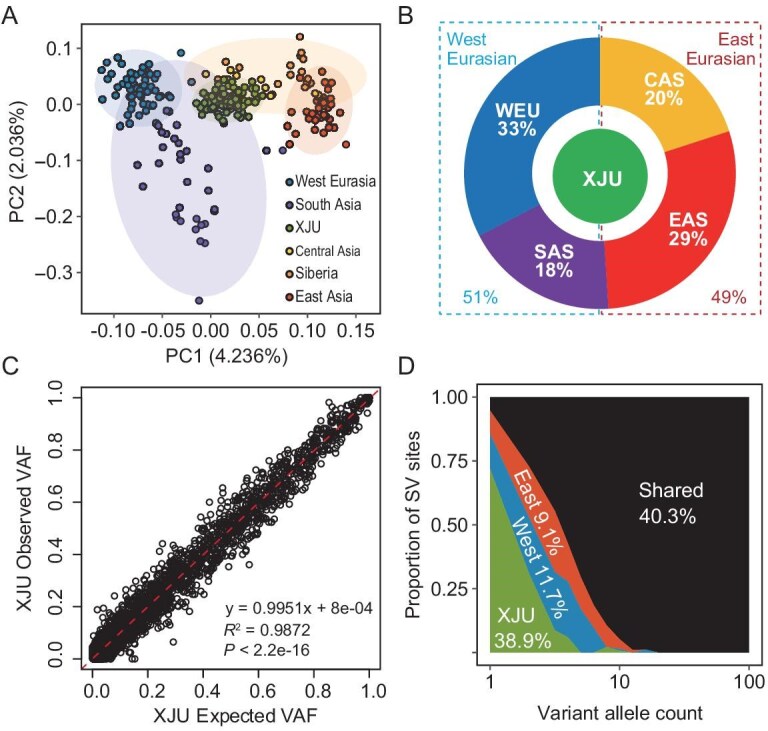
Population genetic properties of XJU SVs. (A) PCA plot of XJU and reference populations from SGDP using deletions. (B) Admixture proportions of XJU samples inferred from deletions. EAS, East Asia; SAS, South Asia; CAS, Central Asia Siberia. (C) Correlation between the expected and the observed VAF of XJU SVs. Each circle denotes an SV. The red dashed line represents the linear regression line. (D) Sharing proportion of CNVs between XJU and ancestral reference populations (see Supplementary data).

We investigated the population structure of XJUs by using SV data combined with SGDP samples. Principal component analysis (PCA) confirms a typical admixture pattern, in which the XJU population was surrounded by the ancestral source populations (ASPs) of Eurasian populations and clustered with the Central Asia samples (Fig. 2A). This pattern was also consistent with our previous study using SNV data [27]. We estimated the genetic contribution from ASPs by using a linear model. The inferred ancestry-proportions were 0.290, 0.184, 0.326 and 0.200 for East Asia, South Asia, West European and Central Asia Siberia, respectively (Fig. 2B); 0.510 for the WEU proportion (the sum of West European and South Asia proportions) and 0.490 for the East Eurasian proportion (the sum of Central Asia Siberia and East Asia proportions) were very close to the estimation of the SNV data (0.512 vs. 0.488 WEU vs. East Eurasian) [27]. When using SGDP WEU and HAN populations as ASPs, a typical two-ancestry admixture could still be observed (Fig. S5), and the expected XJU VAF showed strong agreement with the real VAF in the XJU samples under the two-ancestry model (Pearson’s r = 0.9872, *P* < 2.2 × 10^−^^16^; Fig. 2C). In light of these results, we mainly applied this two-ancestry admixture model in the downstream analyses.

To further quantify the degree of SVs shared with ancestral populations, we conducted an analysis by matching sample sizes and weighting the admixture proportions. The results indicated that a lower bound estimation of 61.1% of XJU CNVs were derived from an ASP, i.e. 38.9% of XJU CNVs were not found in either West or East Eurasian ancestries (Fig. 2D). This estimate aligns closely with our previous findings using microarray data [28] but is nearly 2-fold higher than the proportion of the SNVs specific to XJUs (Fig. S6). This discrepancy is likely attributable to the higher mutation rate of CNVs compared to SNVs. When comparing the sharing patterns between the two ancestries, both the CNV and SNV reveal a higher fraction of shared variants between XJUs and WEUs (Fig. 2D and Fig. S6). This observation is consistent with the admixture proportion inference, which suggests a slightly greater genetic contribution from WEU than East Eurasian sources in the XJU gene pool. Additionally, this could be partially explained by the more diverse sampling of WEU populations compared to HAN populations (Fig. S5).

### Highly deviated SVs from admixture proportion

Despite the global VAF following an expected admixture pattern at the whole-genome scale, the local agreement between the observed and the expected frequencies varies from variant to variant. The SVs with large deviated frequencies could be a signature of natural selection in the admixed population [30]. Here we applied ancestry-biased F-Statistics (F_ST_) to measure the deviation of the observed VAF from the expected VAF for each SV locus (see Supplementary Materials and Methods section). Considering that genetic drift might also account for large ancestry-biased F_ST_, we determined the highly deviated ancestry-biased F_ST_ threshold by conducting simulation based on a two-wave admixture model under a neutral scenario (Fig. S7). Ninety-nine highly deviated SVs were identified from the expected admixture proportion under a significance level of 0.01 (simulated ancestry-biased F_ST_ = 0.0224; Table S2; Fig. S8). Interestingly, some highly deviated SV overlapping genes were associated with insulin and diabetes. For example, the VAF of a 3.4-kb duplication (chr4: 99 813 707–99 817 113) in XJU samples (0.024) was only about one-sixth of the expected VAF (0.138) at the intron of *EIF4E*, which is a typical case with an excessive East Eurasian ancestry proportion, for the duplication is WEU-specific and absent in East Eurasian samples (Table S2). This gene encodes the protein of a component of the eukaryotic translation initiation factor 4F complex, which functionally involves the initiation phase of mRNA translation under high glucose and insulin conditions [31]. Another example is a 1.2-kb deletion at *ADGRL3*, of which the XJU VAF (0.059) was also lower than expected (0.150), which is the case with an excessive WEU ancestry proportion, as the deletion is East Eurasian-specific (Table S2). A recent study found that the distinct splice variants of this gene decrease insulin secretion from pancreatic β-cells [32]. Furthermore, we identified a 1-kb intronic deletion of *RPH3A*. The expected VAF of this deletion in XJU samples (0.059) should have been close to both of the ancestry populations (0.057 in HAN and 0.061 in Europeans), but its observed VAF in XJU samples was 2-fold larger (0.145). The protein encoded by this gene is a secretory vesicle protein rabphilin-3, which might stimulate insulin release [33].

Recent positive selection could cause extended haplotype homozygosity (EHH) near the selected allele due to selective sweep [34], so we tested whether any of the highly deviated SVs in the XJU population also showed strong EHH. We combined SNV data to perform analyses of integrated haplotype score (iHS) [35] and cross-population EHH (XP-EHH) against the ancestral populations [36] for each SV in XJU samples. After excluding the SVs presenting in the top 5% iHS of the West or East ancestral populations, which might be largely due to pre-admixture selection, we identified 21 candidate SVs (|Z-score| > 2) in the XJU samples harboring longer homozygous SV haplotype compared to the non-SV haplotype (Table S3), and 3 of the variants were among the highly deviated SVs. For example, a 5.1-kb intergenic deletion (chr5:143 181 043–143 186 147) at around 5 kb upstream of a minor histocompatibility antigen-encoding gene *HMHB1* (*HLA-HB1*) overlapped several enhancer signals. The observed VAF was 10-fold higher than that expected, and we even observed a homozygous deletion carrier, which was almost 5-fold higher than expected given that the expected homozygous deletion frequency was only 0.003 under Hardy–Weinberg equilibrium in our 85 XJU samples (Fig. S9). In addition, we also observed 10 highly deviated SVs in XJU samples showing XP-EHH signals (|Z-score| > 2), which overlap genes including *GFRA2, PPP2R2B, CLSTN2* and *GALNTL6* (Table S3).

Furthermore, we wanted to know whether there were any highly deviated SVs originally from archaic hominids. Since the introgression from archaic hominins to modern humans occurred about 30 000 years before the present [37], the archaic DNA segments in the XJU genome are most likely to be inherited from ancestral Eurasian populations. To identify the SVs that were originally introgressed from archaic hominids, we used SNVs to locate the introgressed segments in the XJU genomes and screened for the CNVs within the introgressed segments, of which the genotypes were highly correlated with archaic introgressed copies across all the XJU samples, and the variant can be found in the archaic genomes (Pearson’s correlation r^2^ ≥ 0.8; Supplementary data). A total of 37 CNVs were identified as archaic origin variants (Table S4), among which 5 variants were from Denisovans and 32 CNVs were from Neanderthals (including Altai and Vindija Neanderthals). Only one archaic-origin SV was among the highly deviated SVs. Notably, although not passing the threshold of the highly deviated SVs, one Denisovan-origin deletion also has a large ancestry-biased F_ST_ (0.0215), and this variant overlaps the intron of a gene of the neurexin family, *CNTNAP2*.

### Functional implication of SVs in the XJU population

To analyze the potential functional impacts of the XJU SVs, we first categorized the SVs according to their positions relative to the functional elements. Consistent with a previous study [17], we found that the SVs in the CDS and UTR regions were significantly depleted (*P* < 0.001; Fig. S10), and also the common (VAF > 0.1) and moderate (0.01 < VAF ≤ 0.1) deletions were much more depleted than the rare deletions (VAF < 0.01; Fig. S10). Instead, we found that almost 70% of the SVs overlap with at least one ENCODE element [38]. These observations suggest a potential regulatory role of the SVs rather than the impact of protein-coding change.

**Figure 3.**
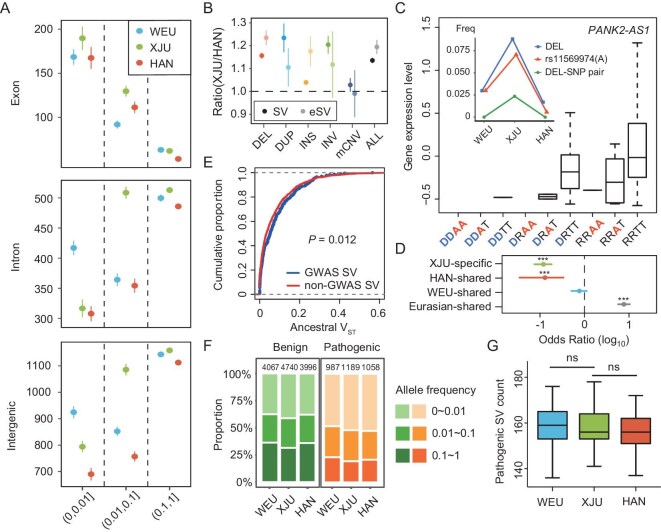
Functional impact of XJU SVs. (A) Overview of SV counts in WEU (blue), XJU (green) and HAN (red) populations; *n* = 50 for each population. The SVs were divided into allele frequency ranges: 0–0.01, 0.01–0.1 and 0.1–1 (*x*-axis), and categorized as exonic, intronic or intergenic (*y*-axis). Each dot and each bar represent the mean and the standard deviation of the SV numbers of 100 random samplings. (B) SV and eSV diversity comparison between XJU and HAN populations. The ratio was calculated as the SV (dark color) and eSV (light color) number in XJU samples divided by the number in HAN samples, respectively. XJU samples were randomly selected from 38 individuals to match the HAN sample size, from which the gene expression data were available. Each dot and each bar represent the mean and the standard deviation of 100 random samplings. (C) *PANK2-AS1* expression is associated with a combination of ancestral rare/low-frequency SNV–SV pairs in the XJU samples. The inset graph shows the allele frequency for the SNV (red) and deletion (DEL; blue) allele and the genotype frequency of the two variant carriers (green). D, deletion allele; R, reference allele; A, SNV rs11569974(A) (GRCh37 alternative) allele; T, SNV rs11569974 (T) (GRCh37 Reference) allele. (D) Enrichment of XJU GWAS-associated SVs in different sharing categories. Points represent the log_10_-scaled odds ratios of GWAS-associated SVs in the particular category versus those in the other categories. Bars represent the 95% CIs. The GWAS-associated SV was defined as the SV with a strong linkage (r^2^ ≥ 0.8) of at least one GWAS SNV in XJU samples. (E) Ancestral V_ST_ comparison between GWAS-associated SV (blue) and non-GWAS-associated SVs (red) in the Eurasian shared category. The Wilcoxon test was used in the comparison of the cumulative distribution of V_ST_. (F) Allele frequency distribution of putative benign and putative pathogenic SVs. The numbers above each bar represent the total SV loci in the corresponding population. (G) The number of putatively pathogenic SVs in each individual genome. ns, not statistically significant.

To compare the potential functional impacts of SVs between the XJU population and its ancestries, we controlled the sample size for each population and counted the number of SVs located at different allele frequency bins in the exon, intron and intergenic regions. We found that the XJU population outnumbered the two ancestral reference populations for the moderate and common SVs, while for the rare SVs the number in the XJU population was in between the two ancestral reference populations at intronic and intergenic regions (Fig. 3A).

Next, we explored the regulatory impact of SVs by conducting expression quantitative trait loci (eQTLs) analyses for gene–SV pairs within cis-regions (defined as 1-Mb intervals). A total of 1542 and 988 eQTL-SVs (eSVs) (adjusted *P* < 0.05; Methods) were detected in 83 XJU and 38 HAN samples, respectively. The analysis revealed distinct regulatory effects across different SV types. For example, insertions exhibited both up- and down-regulation of cis-gene expression, whereas deletions and duplications predominantly showed uni-directional effects, with deletions typically decreasing and duplications increasing cis-gene expression, respectively (Fig. S22).

To gain more insights into the functional significance of eSVs, we compared the allele frequency spectrum of SVs with that of the synonymous and non-synonymous SNVs. The SVs that intersected the entire or partial cis-eQTL genes show a larger proportion of rare variants than the SNVs in the same genes (Fig. S23). We further divided the SVs into four groups based on the overlap between SVs and genes: intergenic, intron, partial gene and whole gene. For the non-coding (intergenic or intron) SVs, the eSV proportion was 21%–22%, while for the coding (partial or whole gene) SVs, the eSV proportion increased to 35%–40% (Fig. S23). Consistently, the effect size of non-coding SVs was significantly lower than that of the exon-overlapped SVs (Fig. S23). These results indicate the large functional impact of coding SVs, and they are under strong selective pressure.

As the sample size would influence the eQTL results, we downsampled XJU samples to match the HAN sample size. This resulted in an average 1.19-fold increase [95% confidence interval (CI): 1.185–1.196] of the cis-eSVs in the XJU population compared with the HAN population, which is even higher than that of the total SVs between XJU and HAN populations (mean: 1.135; *P* < 2.2 × 10^−16^, *t*-test; Fig. 3B), indicating that the increased genetic diversity also elevates the transcriptomic diversity in the admixed population with an even larger fold-change. To further evaluate the relative contribution of SVs, we added flanking biallelic SNVs (i.e. within a 1-Mb region) and partitioned the heritability of gene expression by using a linear mixed model with a fixed effect for SVs and a random effect for SNVs. The joint analysis identified 16 879 eQTLs with an overall genetic heritability (>0.05), among which 4983 (29.4%) cases could be attributed to lead-SV eQTLs, i.e. SVs contributing more than the effect of additive SNVs (Fig. S11).

Previous studies have revealed that the rare variants had a larger impact on gene expression than the common variants [39]. Taking advantage of the whole-genome sequencing data of the XJUs, we investigated the regulatory impact of the ancestral rare/low-frequency SVs, defined as the SVs with VAF < 0.05 in the present-day ASPs. Nearly half (3544/7508) of the rare/low-frequency SVs in the ancestral populations could be observed in the XJU samples, among which 3490 SVs remained at rare/low frequency (VAF < 0.05) in the XJU populations. A total of 935 ancestral rare/low-frequency SVs, including 10 variants with VAF > 0.05, were significantly associated with cis-gene expression in the XJU population (adjusted *P* < 0.05). Given a larger regulatory impact of rare SVs compared with rare SNVs [6], we further investigated whether a gene could be simultaneously up- or down-regulated by two ancestral rare/low-frequency SVs. In our XJU samples, we didn’t find such pairs of SVs, possibly attributed to the deleterious consequence of the additive effect, but we still caution that other factors, such as the stringent SV filtering, sample size and the tissue we analyzed might affect the results. When we focused on the additive regulation impact of the ancestral rare/low-frequency SNV and SV combinations, we observed that eight such pairs resulted in a consistent up- or down-regulation effect (adjusted *P* < 0.05). For example, *PANK2-AS1*, an antisense long non-coding RNA (lncRNA) to *PANK2* (a gene coding the pantothenate kinase, which is a key regulatory enzyme in the biosynthesis of coenzyme A), was found to be down-regulated by either the deletion (chr20: 3 821 167–3 825 155) or the SNV [rs11569974(A)] (Fig. 3C). Interestingly, both the deletion allele and the rs11569974(A) frequency in XJU samples were much higher than for either of the ASPs, especially for the deletion, which was among the top differentiated SVs that deviated from the expected frequency with excessive WEU ancestry (Table S2). Furthermore, the number of samples carrying both alleles was more than expected and less likely explained by sampling error or genetic drift based on our simulation analysis under a two-way admixture model (*P* = 0.001; Supplementary data), suggesting the influence of either demographic events or natural selection on these down-regulated variants of *PANK2-AS1*.

The SNVs curated from the genome-wide association studies (GWAS) catalog can serve as a bridge to associate the SVs with phenotypic traits using the LD information. Here we identified 568 SV-GWAS SNV pairs that were in strong LD (r^2^ ≥ 0.8) in XJU samples. Among these, 88.4% of the pairs were also in strong LD in either of the ancestral populations, suggesting that the XJU population may inherit these traits from the ancestral populations if the GWAS-associated SVs are functional. For example, a height-associated GWAS SNP, rs11950938, was in complete linkage with an insertion at the intron of *NSD1* [40]. We further partitioned the SVs in LD with GWAS according to the sharing relationship into four categories: XJU-specific, HAN shared, WEU shared and Eurasian shared. As expected, the XJU-specific group showed depletion of GWAS-associated SVs (*P* < 2.2 × 10^−16^). On the contrary, GWAS-associated SVs were enriched in the Eurasian shared group (Fig. 3D). Even matching the frequency with the other categories, the strong enrichment of GWAS-associated SVs in the Eurasian shared group still existed (Fig. S12). These results suggest that the ancient Eurasian-shared SVs disproportionately contribute to the phenotypic traits in the XJU population, while there is a potential ascertainment bias in the discovery of GWAS findings, which makes the GWAS signals largely unexplored in the non-European populations. Furthermore, we used V_ST_ [41] To indicate the ancestry differentiation. A large V_ST_ value represents a large differentiation between two ancestral populations. The cumulative V_ST_ distribution showed that GWAS-associated SVs had a larger West–East Eurasian differentiation compared to the non-GWAS-associated SVs (*P* = 0.012, Wilcoxon test; Fig. 3E), indicating that the GWAS-associated SVs might be favored by natural selection during population divergence.

To assess the medical implications, we annotated the clinical impact of SVs using AnnotSV [42]. Most putative pathogenic SVs tend towards lower frequency compared with the putative benign SVs (Fig. 3F). Interestingly, despite excessive total SVs for each individual genome (Fig. 1C, Fig. 3A and B), the number of the putatively pathogenic SVs in the XJU population is in between that of the two ASPs with no significant difference from either of the ancestral populations (Fig. 3G). These results are also in line with the total number of rare variants of the admixed population, being in between that of the two ASPs (Fig. 3A), suggesting that the genetic burden of SVs might not get increased as the overall SV diversity increases in the admixed population.

### Admixture-induced genetic diversity and functional impact

**Figure 4.**
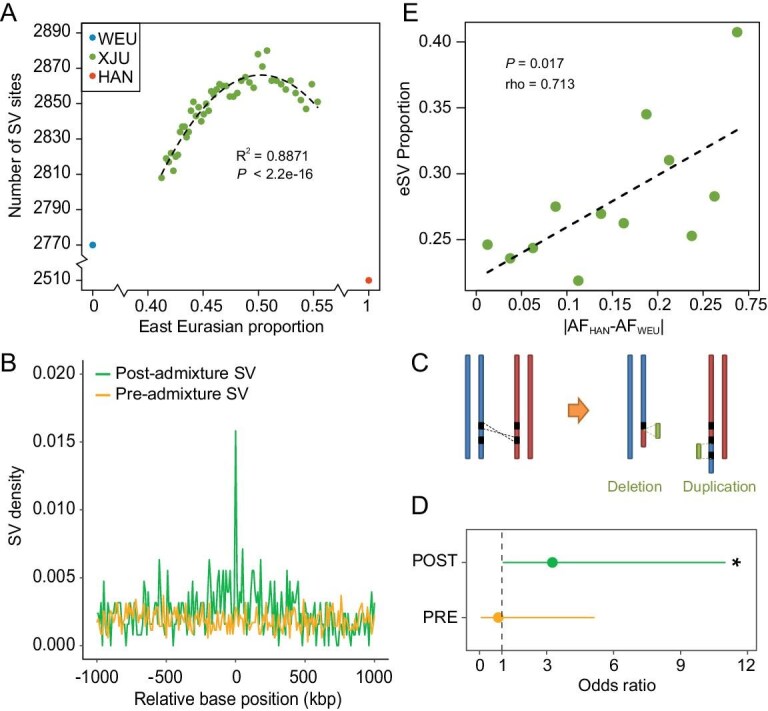
Admixture pattern of genetic and transcriptomic diversity. (A) Relationship between SV diversity and ancestry proportion. The SVs in this plot did not include duplication, see Fig. S14 for all the SV types. Each green dot represents the number of SV sites from a sampling of 40 XJU individuals. The blue and red dot represent the mean number of SV sites from 100 samplings from 40 WEU and HAN individuals, respectively. The dashed line represents the fitted parabola curve. *R*^2^ represents the correlation using the parabola fitting model. (B) The density plot of SVs with the distance from the nearest ancestry-switch point. (C) Illustration of inter-ancestry NAHR causing SVs. Red and blue bars represent East Eurasian and WEU ancestry chromosomes, respectively. Green bars represent the SVs in the XJU population. (D) Enrichment of breakpoint homology for the SVs overlapping the ancestry-switch point. Orange and green indicate the pre- and post-admixture SVs, respectively. * corresponds to *P* < 0.05 (two-sided Fisher’s exact test). Bars represent 95% CIs. (E) Relationship between eSV proportion and ancestral allele frequency (AF) difference. Each point represents the eSV proportion in each bin of the absolute allele frequency difference between two ancestries with a bin size of 0.025, except the last bin, which has a range of 0.3–1. The dashed line shows the fitted line, and rho is Spearman’s correlation coefficient.

To investigate the pattern of how the ancestry proportion affects the SV diversity in the admixed population, we proposed two possible models: one is a linear model in which the genetic diversity of the admixed population grows as the proportion of the ancestry with larger diversity increases; the other one is a non-linear model in which the genetic diversity and the ancestry proportion follow a parabola curve (Fig. S13). To test the hypothesis, we analyzed the SV segregating sites in sequential *in silico* XJU subpopulations with a monotonically increasing East Eurasian proportion (Supplementary Materials and Methods). In these XJU subpopulations, we observed that the number of segregated SVs supported a quadratic model (*R*^2^ = 0.887) better than the linear one (*R*^2^ = 0.512) in all SVs excluding duplications (Fig. 4A; Fig. S14). The genetic diversity in any subpopulation was higher than that in either of the ancestral populations and reached the maximum when the two ancestry proportions were close to being equal (Fig. 4A). The permutation test showed that the parabola-like pattern was less likely explained by chance (*P* = 0.002; Fig. S15), indicating that the SV diversity of an admixed population is largely determined by the ancestry proportion and follows a quadratic model.

We then assessed whether the admixture process would induce SVs, i.e. the occurrence of the SVs caused by the inter-ancestry recombination events. To test this, we categorized the pre- and post-admixture CNVs by using a stringent sharing strategy, and calculated the SV density with respect to the ancestry-switch point (Supplementary Materials and Methods). Compared to the pre-admixture CNVs, which followed a uniform density distribution, the post-admixture variants were enriched at the ancestry-switch point (Fig. 4B). The permutation test with shuffling haplotype and SVs confirmed the enrichment was less likely observed by chance or by phasing error (*P* = 0.037). Other factors such as the length and the frequency of the SVs that might putatively affect the results were also excluded (Fig. S16), which confirmed that a certain proportion of SVs were induced during the admixture events and indicated the ancestry-switch point as a hotspot for the post-admixture SVs. As these SVs emerged with ancestry-switch events, they were most likely induced by NAHR (Fig. 4C). To test this hypothesis, we searched for the homology flanking the breakpoints of the SVs. The results showed that the post-admixture SVs at the ancestry-switch points were significantly enriched with flanking homology compared with other post-admixture SVs (*P* = 0.0297), while the pre-admixture SVs were not (*P* = 1, Fig. 4D). Taken together, these results indicated that inter-ancestry recombination would cause SVs via NAHR (Fig. 4C) and the ancestry-switch point could be one of the SV hotspots in the admixture population.

Next, we attempted to address whether the extent of the SV ancestry divergence was related to gene regulation in the admixed population. By analyzing the eSV proportion in each allele frequency difference bin, we observed a positive correlation between the ancestry divergence and the proportion of eSVs (Spearman’s rho = 0.7132867; *P* = 0.01714; Fig. 4E). This suggests that SVs with large ancestry differentiation are more likely to be functional in the admixed population, which is also consistent with the result that the GWAS-associated SVs in the XJU genome tend to have larger differentiation between the two ancestral populations (Fig. 3E). These observations lay the biological foundation for the admixture mapping as such a disease or trait-mapping strategy relies on the DNA segments with large ancestry divergence.

### XJU-specific combinations of genetic variants

**Figure 5.**
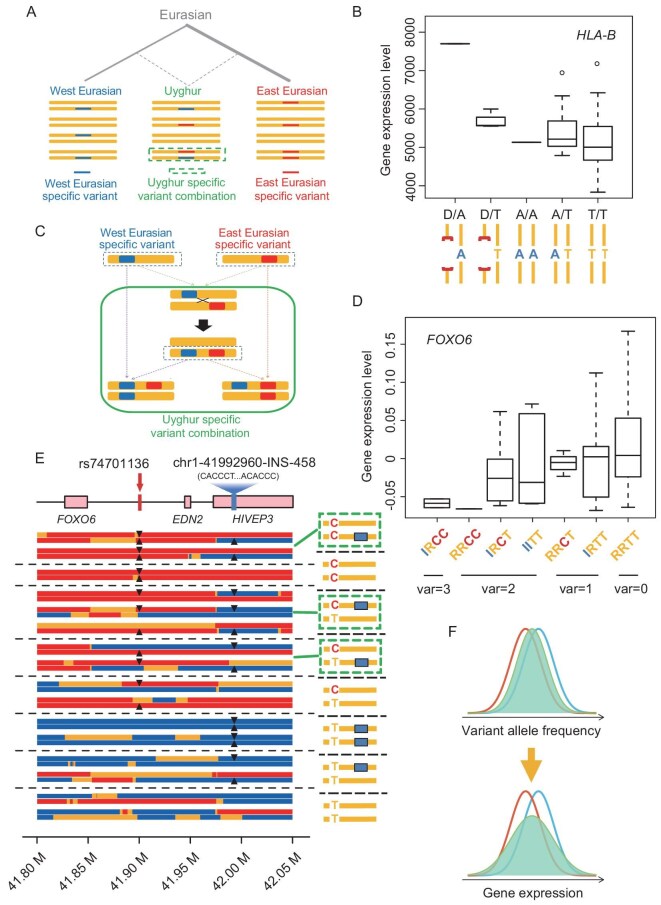
XJU-specific variant combination. (A) Illustration of XJU-specific variant combination at the same locus. Each orange bar represents a haplotype that emerged before the admixture. The blue and red boxes represent the West and the East Eurasian-specific variants that emerged on the lineage before admixture, respectively. The dashed green box indicates the XJU-specific variant combination, i.e. the XJU individual genome harbors both the West and the East Eurasian-specific variant. (B) The *HLA-B* expression level for different genotypes. D, deletion (East Eurasian-specific; red square brackets); T, SNV T-allele (reference allele; orange); A, SNV A-allele (WEU-specific; blue). In this case, genotype ‘D/A’ is the XJU-specific variant combination. (C) Illustration of XJU-specific variant combination through recombination at two loci. Recombination occurs between the West (blue) and the East (red) Eurasian-specific variants. As a result, a haplotype containing both the West and the East Eurasian-specific variants emerges. This recombined haplotype together with other haplotypes containing ancestry-specific variants forms the class of XJU-specific variant combinations (green box). (D) *FOXO6* expression level for different genotypes. I, insertion allele (chr1-41992960-INS-458; WEU-specific; blue); R, reference allele without insertion; T, rs74701136 reference allele (orange); C, rs74701136 alternative allele (East Eurasian-specific; red). In this case, genotypes IRCC and IRCT are the XJU-specific variant combination. (E) Local ancestry inference of XJU representative haploid combinations at the *FOXO6* region. Each row on the left panel represents a haploid with inferred ancestry (blue, WEU; red, East Eurasian; orange, common Eurasian) from ChromoPainter [64], and every two haploids separated by a blank row represent a haploid combination in the XJU samples. Dashed black lines separate different genotypes. Genes are shown in the pink box at the top. The haplotypes on the right panel are the same as in (D). The green dashed box indicates the XJU-specific variant combinations. For display purposes, we only show one or two individual genotypes for each genotype combination; see Fig. S18 for genotypes of all 85 XJU samples. (F) Diagram of the relationship between genetic diversity and transcriptomic diversity in the admixed population. For most SV loci, the allele frequency distribution of the admixed population is in between the two ancestral populations (upper panel); however, the gene expression range of the admixed population could be much larger than the two ancestries due to the combined effect of the genetic variants in the admixed population (lower panel) for certain genes. See details in Fig. S19.

After migrating out of Africa, the ancient Eurasian population split and diverged during the subsequent 45 000 years [43,44], which enabled the accumulation of population-specific genetic variants in the West and the East Eurasians. These specific variants were retained with a certain frequency in each population, but they could be hardly found in one gene pool or emerged in a single individual without gene flow. However, owing to admixture, the XJU population harbors both West and East Eurasian-specific variants in its gene pool, which makes the two specific variants presenting in one single individual possible (Fig. 5A). For example, XJU samples carry both an East Eurasian-specific deletion and a WEU-specific SNV at the deletion region of the *HLA-B* locus (Fig. 5B; Fig. S17). We termed the genotype of combining ancestry-specific alleles as XJU-specific variant combination (USVC). In our 85 XJU samples, we found 519 USVCs that contained one CNV with at least one overlapping SNV (DEL–SNV or DUP–SNV pair), related to 251 different SVs presenting in at least one XJU individual. We further jointly analyzed the functional impact of these CNV–SNV pairs on gene expression using a linear model, and identified 39 DEL–SNV pairs that were significantly associated with gene expression (adjusted *P*_DEL_ < 0.1 and adjusted *P*_SNV_ < 0.1; Table S5), while no significant DUP–SNV pairs were found. For the example of the *HLA-B* locus, the USVC with the combination of a WEU-specific SNV T-allele (rs3131623) and an East Eurasian-specific deletion allele (chr6: 31 357 451–31 454 440) had the lowest expression level of the important immune-related gene *HLA-B* among all the genotypes observed in our data (Fig. 5B). Consistently, the sequence overlapping the deletion (and the SNV) was annotated as repressed chromatin state [45] (Fig. S17), and the lowest expression was likely explained by the additive effect of the two down-regulated variants.

The USVCs could also be extended to the case of one ancestry-specific SV and the other ancestry-specific SNV located within the 1-Mb flanking region of the SV position (cis-SV–SNV pair). As the SNV is not required to be overlapping the SV site, there could be recombination between the SV and SNV, which further increases the haplotype diversity (Fig. 5C). In our 85 XJU samples, there were 1 487 788 cis-SV–SNV pairs related to 2971 different ancestry-specific SVs presenting in at least one XJU individual. Again, using a regression model, we found 137 cis-SV–SNV pairs that both SV and SNV were significantly associated with the gene expression (adjusted *P*_SV_ < 0.1 and adjusted *P*_SNV_ < 0.1; Table S5). For example, a WEU-specific 458-bp insertion at *HIVEP3* and an East Eurasian-specific SNV (rs74701136; T > C) at the downstream region of *FOXO6* were both associated with the expression level of *FOXO6*, which integrates insulin signaling with gluconeogenesis in the liver [46]. The insertion was enriched in WEU samples with a VAF of 0.440 in contrast to 0.012 in HAN samples, while the alternative SNV allele C was enriched in HAN samples, with a VAF of 0.225 in contrast to 0.015 in WEU samples. The combination of C-allele and insertion-allele in one single genome could be observed in XJU samples (Fig. 5D). Even more, we observed that two XJU samples carried a homozygous C-allele and a heterozygous insertion, which indicates that at least one recombination event happened between the insertion site and the SNV locus. The local ancestry inference confirmed that the XJU-specific configuration samples carried an inter-ancestry recombined haplotype at the two sites (Fig. 5E; Fig. S18). Because of the dual down-regulated effect of the insertion allele and the SNV C-allele, samples with more variant alleles were expected to have lower expression levels of *FOXO6* compared to those with fewer variant alleles. Since the inhibition of *FOXO6* was found to attenuate hepatic gluconeogenesis in the insulin-resistant liver of diabetic mice [46], the lower expression of *FOXO6* might play a protective role against hyperglycemia and glucose intolerance in XJU individuals.

In addition to the cis-SV–SNV pairs, we further identified 2187 cis-SV–SV pairs following the USVC model, but we didn’t find any combinations that were significantly associated with gene expression. Taken together, we identified 176 USVCs associated with gene expression, in which 33.5% of the combinations belonged to the case of the two variants with the same direction of regulatory effect. These results demonstrated that the combination of ancestry-specific genetic variants produced a unique genetic combination and induced an admixture-specific expression pattern in the admixed population, suggesting that not all the molecular function/trait levels in the admixed population are expected to be in between the ASPs, but could also go beyond the spectrum of ASPs, in principle, with a larger impact and a wider range (Fig. 5F; Fig. S19).

### Evolutionary patterns and implications of SVs in the XJU genome

**Figure 6.**
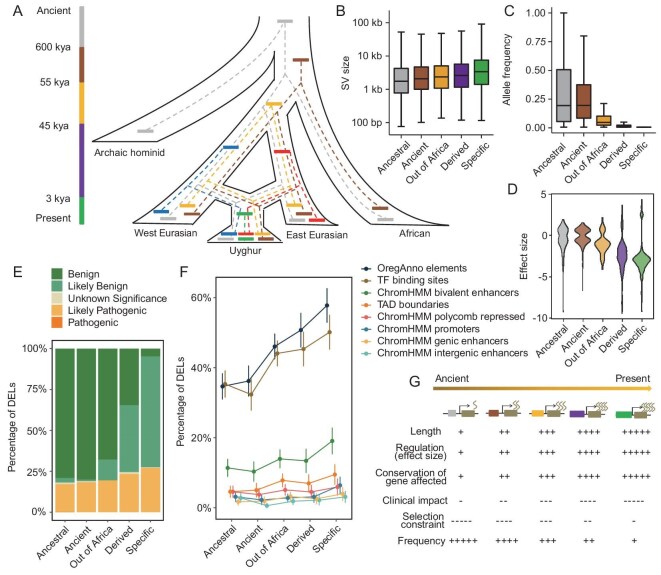
Evolutionary patterns of SVs of different ages in the XJU genome. (A) Phylogeny of SVs of different ages. Each colored rectangle denotes an SV corresponding to the color, with the time scale on the left axis, which represents the time that the SV first emerged. According to the time scale, the XJU SVs were divided into five groups: the ancestral group (gray), the ancient group (brown), the Out-of-Africa group (orange), the Eurasian-derived group (purple on the left axis; red and blue rectangles), and the XJU-specific group (green). Note that the time axis is only for display purposes; its scale is not proportional to the real time. (B) SV size distribution among different groups. (C) SV allele frequency distribution among different groups. (D) Effect size of eSV distribution among different groups. Gene expression was Z-score-normalized across all genes. (E) Clinical impact of SVs inferred by AnnotSV [42] among different groups. (F) Proportion of SVs disrupting functional elements among different groups. TF, transcriptional factor; TAD, topologically associated domain. (G) Summary of evolutionary properties for SVs of different ages. The box in champagne represents a gene, and the box with other colors identical to (A) represents an SV. The rotated wavy lines represent the mRNA. + represents a positive effect and − represents a negative effect.

The XJU genome comprises the SVs that not only came from the ancestral populations but also emerged recently after the admixture event. Since the genetic make-up and the genetic history are relatively clear, using XJUs as a model population could help us understand the signatures and the evolutionary properties of SVs that emerged at different time scales, especially those young variants. Based on the phylogeny, we divided the XJU SVs into five classes using a sharing approach (Fig. 6A): (i) the ancestral group, representing the variants that emerged before the divergence between the archaic hominids and the modern human; (ii) the ancient group, representing the modern human-specific variants; (iii) the Out-of-Africa group, denoting the variants that emerged during the modern human Out-of-Africa period; (iv) the derived group, representing the variants that occurred after the divergence between the West and East Eurasians; and (v) the XJU-specific group, denoting the recent and the putative post-admixture events. We analyzed the properties of XJU deletions across these five classes to explore the evolutionary patterns of the SVs. We found that the size of the deletion was significantly correlated with the variant age, i.e. a variant with younger age tended to have a larger size (*P* = 0.001, Jonckheere’s trend test; Fig. 6B), while an inverse correlation pattern was observed between the allele frequency and the deletion, i.e. a younger variant tended to be less frequent in the population (*P* = 0.001, Jonckheere’s trend test; Fig. 6C). We further analyzed the impact on gene regulation by comparing the effect size for the eSVs at different lineages. We observed that the effect size also significantly correlated with the age of the SV, i.e. a younger variant tended to have a greater impact at the transcription level (*P* = 0.001, Jonckheere’s trend test; Fig. 6D), which was consistent with the trend of the SV size, as a larger variant covered more regulatory elements, which might, in turn, cause a larger effect (Fig. 6F). Furthermore, the conservation of both the SV intersected genes and the eSV regulated genes were significantly associated with the variant age (*P* < 0.005, Jonckheere’s trend test; Fig. S20), in which the genes affected by young SVs are relatively more conserved than those affected by ancient SVs. Due to the greater molecular functional impact for the younger SVs compared with the older ones, we hypothesized that the younger SVs might have a larger phenotypic impact as well. The clinical impact inferred by AnnotSV confirmed that the younger age variants were more likely to be pathogenic than the older ones (Fig. 6E). All these patterns indicate that constraint selection may shape the size, frequency and impact of SVs in the present-day genomes, especially acting strongly against the commonly shared SVs that persist for a long time in the human population, but are relaxed or inefficient for the new mutations (Fig. 6G).

The admixture nature of the XJU population enables us to further study the post-admixture SVs that emerged recently in evolution. Around 95% of the post-admixture variants were singletons. As we showed above that the frequency and the size of the SVs were correlated with the functional impact, we focused on singletons and conducted the comparative analysis between the pre- and post-admixture variants by controlling SV length. The results showed that the post-admixture deletions were significantly more likely to affect gene expression, and with a larger effect size than the pre-admixture variants (*P* < 0.01; Fig. S21). Furthermore, the genes affected by post-admixture eSVs were more conserved than those affected by pre-admixture eSVs, which is consistent with the clinical impact prediction that the post-admixture deletions were predicted to be less benign and more pathogenic than the pre-admixture deletions (Fig. S21). All these findings indicate that even when controlling the size and frequency in the XJU population, the post-admixture SVs still have larger functional impacts compared with the pre-admixture SVs, which further suggests the biomedical importance of these young SVs in the admixed population.

## DISCUSSION

In this study, we investigated the structural variation of a model admixed Eurasian population with distinct Western and Eastern ancestral components using whole-genome sequencing. By conducting population structure and simulation analysis, we confirmed that the global SVs in the XJU genomes followed the admixture model with almost identical ancestry proportions as when estimated by SNVs. However, there are discrepancies in the patterns uncovered between SV and SNV data. For example, SVs exhibit a 2-fold greater XJU-specific variant proportion compared with SNVs, indicating variants with distinct mutation rates have different genomic signatures on a population level. These XJU-specific SVs comprise more than one-third of the segregating SVs and most of them are singletons, which have greater functional and clinical impacts compared with other old SVs. These XJU-specific SVs could hardly be captured in the admixture mapping as such methodology mainly relies on the linkage between the ancestry and the disease [47], suggesting the necessity of a comprehensive SV analysis for admixed populations such as the XJU population when conducting genetic and medical studies.

The previous association studies have identified a few SNVs associated with traits, and most of them were based on microarray data. Using LD between SVs and GWAS SNVs, we can establish the relationship between SVs and traits. As expected, the GWAS-associated SVs are depleted in the XJU-specific group, due to not only the ascertainment bias of the microarray but also the fact that the Eurasian admixed populations have been underrepresented in association studies. In addition, we observed that several highly deviated SVs from the expected admixture proportion were putatively associated with insulin or diabetes, which might provide a genetic basis for future disease studies as the XJU population has been reported to have a higher incidence of type 2 diabetes than other Chinese ethnic groups [48,49]. However, further functional studies are required to investigate the roles of these variants in the XJU population, and also GWAS studies for the admixed populations are required.

It is known that the population admixture could increase the genetic diversity, but how the admixture proportion changes the genetic diversity remains unexplored. By sequentially sampling subgroups with a monotonical admixture proportion, we found a strong correlation between the population admixture proportion and the number of SV segregating sites. Our observation reveals that when the two divergent ancestries mixed with almost equal contribution, the admixed population would have the largest genetic diversity. Such a trend is statistically significant and consistent for other SV types (deletions, insertions, inversions and mCNVs), indicating such a correlation is hardly explained by chance. Interestingly, XJU samples didn’t show significantly increased pathogenic SVs compared with ASPs, indicating that the genetic burden of SVs in the admixed population was maintained at a certain level by natural selection.

Our study provides empirical evidence that ancestry transition zones in admixed populations—which reflect historical recombination breakpoints—represent preferential sites for SV hotspot formation. The most likely mechanism is the NAHR during meiosis, as more than 60% of the SVs at the ancestry-switch point have flanking sequences in homology, which is almost 2-fold higher compared with the genome-wide level (∼32%). The majority of the homologous sequences were attributed to repeats such as LINE and Alu, consistent with the previous findings of LINE–LINE- or Alu-mediated NAHR [50,51].

The admixture would increase not only the genetic diversity but also the transcriptomic diversity. Although the fold-change for the genetic diversity and transcriptomic diversity varies from type to type, the overall transcriptomic increase is more pronounced than the overall genetic increase when comparing the XJU ancestry to the East Eurasian ancestry (Fig. 3B). Further study using WEU ancestry as reference is required to fully understand the transcriptomic increase of the XJU population. When comparing the eSV proportion among different levels of ancestry divergence, the SVs with larger ancestry differentiation are more likely to be associated with gene expression. One possible explanation is that these ancestry-differentiated SVs might be involved in gene function and associated with an adaptive trait, which is supported by the GWAS analysis that the GWAS-associated SVs tend to have a larger ancestral V_ST_. This observation indicates that the admixture mapping is practicable for those ancestry-inherited SVs in the XJU population, and also that the highly differentiated SVs could be the candidate causal variants due to their large size and functional impact.

Another previously unexplored aspect of the genetic diversity of the admixed populations is the effect of the genetic variant combination. Particularly, the combinations of population-specific variants emerge frequently in the admixed individuals. There are many classes of the population-specific variant combinations, such as SV and SNV, SV and STR, SV and INDEL etc. In this study, we mainly focused on SV and SNV combinations and used the USVC of *HLA-B* and *FOXO6* to demonstrate that the combination of the variants may jointly affect gene expression. As shown in the two examples, the two ancestry-specific variants have the same direction of regulation towards gene expression, which causes a more extreme expression level compared with other combinations, indicating that admixture would not only cause the genomic feature of the admixed population, such as the copy-number or allele frequency spectrum, distributed between the two ancestral populations, but may also broaden the transcriptome or phenotypic level in a wider range (Fig. 5F; Fig. S19). Among the USVC associated with gene expressions, one-third of the combinations affect regulation in a uni-directional way. The medical implication for the uni-directional regulation is that the additive down- or up-regulation aggravates the deviation from the normal expression range, which might result in aberrant gene function and eventually affect the phenotype. A typical medical example has demonstrated such a combination effect for the risk of congenital scoliosis, in which a combination of point mutation and deletion would dramatically reduce expression of the *TBX6* gene and in turn cause disease, but the point mutation or the deletion alone would only moderately decrease the gene expression, with no significant consequence on phenotype [52].

The XJU population could also serve as a model population to help understand the evolutionary patterns of SVs in the human genome as it has a relatively clear genetic make-up and genetic history. By dissecting the XJU SVs into different lineages, we demonstrated the signatures of the present-day XJU SVs that emerged at different evolutionary time scales. The pattern implies that the length and the disrupted functional elements may determine the fate of the SVs. Ancient SVs tend to be small in size and with minor or little functional impact in the present-day XJU genome. On the contrary, the recent SVs with a larger size and impact are more likely population-specific, and subject to stronger selection constraints. However, due to the inefficient selection against the recent emerging variants, such large SVs are still observable in the population, albeit in low frequency. The post-admixture SVs are typical examples. These recently emerging variants comprise almost one-third of the total SV segregating sites in the XJU population. A higher priority should be given to the post-admixture SVs, even though they have an identical allele frequency and similar size of pre-admixture event, in the XJU population in the precision medicine and disease study, as such variants are more likely ‘pathogenic’.

We noticed that the duplication in the XJU population shows different patterns compared to other SV types. At the whole-genome scale, duplication could not be as well tagged by flanking SNVs as other SV types (Fig. S4), which is also observed in other studies [4,17]. The admixture proportion and the genetic diversity showed a bell-shape correlation for all the SV types except for duplication, which followed an inverse-bell shape (*R*^2^ = 0.351; *P* = 3.4 × 10^−^^5^; Fig. S14). Considering duplication may have multiple roles in evolutionary history [53], especially some old-aged events involved in the gene duplication of speciation [54], we speculated that the shared duplications in the human genome for a long time might be under positive selection or reduced selection compared with the new duplication, which occurred recently. Similar patterns, with the duplication exhibiting different evolutionary features as a result of distinct mutation rate and evolutionary constraint compared with other SV types, are also reported in other studies [4,16]. However, due to its lower validation rate compared with other SV types (Table S1) we caution that the admixture patterns of duplication in this study might need further inspection.

In summary, this study has further advanced our knowledge of the genetic diversity of an admixed population and also fills the gap of how the admixture as an evolutionary driving force acts on the genetic diversity and the potential functional consequence. Our results uncover the evolutionary patterns for SVs of different ages, which facilitate our understanding of the evolutionary properties and screening for the trait and disease-associated variants.

## MATERIALS AND METHODS

### Sample information

We collected peripheral-blood samples of 92 XJU individuals from the Xinjiang Uyghur Autonomous Region, China, and those of 90 HAN individuals from diverse regions [55,56]. Each individual was a third (or more) generation offspring of a non-consanguineous marriage of members of the same nationality within three generations.

### Genome sequencing and data processing

For both the XJU (*n* = 92) and HAN samples (*n* = 90), whole-genome sequencing was performed on Illumina HiSeq X Ten following Illumina-provided protocols. All samples were whole-genome sequenced to an average depth (∼30×) and 150 bp paired-end reads. Among the total of 182 samples, 7 samples (1 XJU and 6 HAN) were additionally sequenced as replicates that were used as SV-calling quality control in the downstream analysis. In addition, we downloaded the high-coverage (>30×) whole-genome sequencing data of 73 WEU samples from the SGDP, which were paired-end sequenced using Illumina HiSeq2000 [4]. All the sequencing reads of XJU, HAN and SGDP samples were mapped to the human reference genome (GRCh37) with Burrows–Wheeler Aligner [57]. The SNV callings were performed using the Genome Analysis Toolkit (GATK) [58]. We applied multiple algorithms/software to call SVs from the bam files (Fig. S1), which utilize the read information such as read depth (RD), pair-end (PE), and split read (SR). The SV calls from each algorithm were merged to an assembled SV callset. We benchmarked our SV pipeline, and the overall precision for SVs is greater than 0.82 (see details in Supplementary Materials and Methods and Table S1).

For 90 of the 92 XJU samples and 39 out of 90 HAN samples, we also performed RNA sequencing with the Illumina HiSeq2000 platform. The trimmed reads were mapped to the human reference genome (GRCh37) using STAR [59]. The gene expression was quantified and normalized using RSEM [60].

### Statistical and population genetic analysis

We assembled the SV calls from each individual sample and merged the intersecting variants passing the quality control into SV regions. PCA was used to study the population structure, and the admixture proportion was inferred by a linear model. We simulated the genetic drift during the admixture process using AdmixSim [61]. We calculated ancestry-biased F_ST_ [28], iHS [35] and XP-EHH [36] to detect natural selection. The eQTL analysis of gene expression and SV was performed by MatrixEQTL [62] and a modified linear model. The ancestry inference was made by PCAdmix [63] and Chromopainter [64]. Detailed descriptions of the methods are available in the Supplementary data.

### Ethics

All samples were collected with informed consent and approved by the Biomedical Research Ethics Committee of Shanghai Institutes for Biological Sciences (No. ER-SIBS-261408). The personal identifiers of all samples, if any existed, were stripped off before sequencing and analysis. All procedures were in accordance with the ethical standards of the Responsible Committee on Human Experimentation and the Declaration of Helsinki of 1975, as revised in 2000.

## Supplementary Material

nwaf527_Supplemental_Files

## Data Availability

The frequency data are available in Table S2. The genotype data are available upon request. The code for SV calling/genotyping pipeline was deposited at https://github.com/Shuhua-Group/PGGSVpipeline. The release of the genetic data by this work is recorded by the National Health Commission (NHC) of the People’s Republic of China (Approval No. 2022BAT2475). All data generated or analyzed during this study are included in this published article, its Supplementary data files, and publicly available repositories.

## References

[1] Alkan C, Coe BP, Eichler EE. Genome structural variation discovery and genotyping. Nat Rev Genet 2011; 12: 363–76. 10.1038/nrg2958 21358748 PMC4108431

[2] Feuk L, Carson AR, Scherer SW. Structural variation in the human genome. Nat Rev Genet 2006; 7: 85–97. 10.1038/nrg1767 16418744

[3] Langefeld CD, Ainsworth HC, Graham DSC et al. Transancestral mapping and genetic load in systemic lupus erythematosus. Nat Commun 2017; 8: 16021. 10.1038/ncomms16021 28714469 PMC5520018

[4] Sudmant PH, Mallick S, Nelson BJ et al. Global diversity, population stratification, and selection of human copy-number variation. Science 2015; 349: aab3761. 10.1126/science.aab3761 26249230 PMC4568308

[5] Spielmann M, Lupiáñez DG, Mundlos S. Structural variation in the 3D genome. Nat Rev Genet 2018; 19: 453–67. 10.1038/s41576-018-0007-0 29692413

[6] Chiang C, Scott AJ, Davis JR et al. The impact of structural variation on human gene expression. Nat Genet 2017; 49: 692–9. 10.1038/ng.3834 28369037 PMC5406250

[7] Perry GH, Dominy NJ, Claw KG et al. Diet and the evolution of human amylase gene copy number variation. Nat Genet 2007; 39: 1256–60. 10.1038/ng2123 17828263 PMC2377015

[8] Bolognini D, Halgren A, Lou RN et al. Recurrent evolution and selection shape structural diversity at the amylase locus. Nature 2024; 643: 617–25. 10.1038/s41586-024-07911-1 PMC11485256 39232174

[9] Stefansson H, Meyer-Lindenberg A, Steinberg S et al. CNVs conferring risk of autism or schizophrenia affect cognition in controls. Nature 2014; 505: 361–6. 10.1038/nature12818 24352232

[10] Glessner JT, Wang K, Cai G et al. Autism genome-wide copy number variation reveals ubiquitin and neuronal genes. Nature 2009; 459: 569–73. 10.1038/nature07953 19404257 PMC2925224

[11] CNV and Schizophrenia Working Groups of the Psychiatric Genomics Consortium . Erratum: Contribution of copy number variants to schizophrenia from a genome-wide study of 41,321 subjects. Nat Genet 2017; 49: 651. 10.1038/ng0417-651d 28358131

[12] Chen X, Schulz-Trieglaff O, Shaw R et al. Manta: rapid detection of structural variants and indels for germline and cancer sequencing applications. Bioinformatics 2016; 32: 1220–2. 10.1093/bioinformatics/btv710 26647377

[13] Chaisson MJ, Huddleston J, Dennis MY et al. Resolving the complexity of the human genome using single-molecule sequencing. Nature 2015; 517: 608–11. 10.1038/nature13907 25383537 PMC4317254

[14] Wang Y, Ling Y, Gong J et al. PGG.SV: a whole-genome-sequencing-based structural variant resource and data analysis platform. Nucleic Acids Res 2023; 51: D1109–16. 10.1093/nar/gkac905 36243989 PMC9825616

[15] Gao Y, Yang X, Chen H et al. A pangenome reference of 36 Chinese populations. Nature 2023; 619: 112–21. 10.1038/s41586-023-06173-7 37316654 PMC10322713

[16] Ebert P, Audano PA, Zhu Q et al. *De novo* assembly of 64 haplotype-resolved human genomes of diverse ancestry and integrated analysis of structural variation. Science 2021; 372: eabf7117. 10.1126/science.abf7117 33632895 PMC8026704

[17] Sudmant PH, Rausch T, Gardner EJ et al. An integrated map of structural variation in 2,504 human genomes. Nature 2015; 526: 75–81. 10.1038/nature15394 26432246 PMC4617611

[18] Byrska-Bishop M, Evani US, Zhao X et al. High-coverage whole-genome sequencing of the expanded 1000 genomes project cohort including 602 trios. Cell 2022; 185: 3426–40.e19. 10.1016/j.cell.2022.08.004 36055201 PMC9439720

[19] Lei C, Liu J, Zhang R et al. Ancestral origins and admixture history of Kazakhs. Mol Biol Evol 2024; 41: msae144. 10.1093/molbev/msae144 38995236 PMC11272102

[20] Chen H, Xu S. Population genomics advances in frontier ethnic minorities in China. Sci China Life Sci 2025; 66: 961–73. 10.1007/s11427-024-2659-2 39643831

[21] Zhang R, Ni X, Yuan K et al. *MultiWaverX*: modeling latent sex-biased admixture history. Briefings Bioinf 2022; 23: bbac179. 10.1093/bib/bbac179 35598333

[22] Wen J, Liu J, Feng Q et al. Ancestral origins and post-admixture adaptive evolution of highland Tajiks. Natl Sci Rev 2024; 11: nwae284. 10.1093/nsr/nwae284 40040643 PMC11879426

[23] Xu S, Jin L. A genome-wide analysis of admixture in Uyghurs and a high-density admixture map for disease-gene discovery. Am Hum Genet 2008; 83: 322–36. 10.1016/j.ajhg.2008.08.001 PMC2556439 18760393

[24] Pan Y, Zhang C, Lu Y et al. Genomic diversity and post-admixture adaptation in the Uyghurs. Natl Sci Rev 2022; 9: nwab124. 10.1093/nsr/nwab124 35350227 PMC8953455

[25] Xu S, Huang W, Qian J et al. Analysis of genomic admixture in Uyghur and its implication in mapping strategy. Am Hum Genet 2008; 82: 883–94. 10.1016/j.ajhg.2008.01.017 PMC2427216 18355773

[26] Xu S, Jin W, Jin L. Haplotype-sharing analysis showing Uyghurs are unlikely genetic donors. Mol Biol Evol 2009; 26: 2197–206. 10.1093/molbev/msp130 19564211

[27] Feng Q, Lu Y, Ni X et al. Genetic history of Xinjiang’s Uyghurs suggests bronze age multiple-way contacts in Eurasia. Mol Biol Evol 2017; 34: 2572–82. 10.1093/molbev/msx177 28595347

[28] Lou H, Li S, Jin W et al. Copy number variations and genetic admixtures in three Xinjiang ethnic minority groups. Eur J Hum Genet 2015; 23: 536–42. 10.1038/ejhg.2014.134 25026903 PMC4666576

[29] MacDonald JR, Ziman R, Yuen RK et al. The database of genomic variants: a curated collection of structural variation in the human genome. Nucl Acids Res 2014; 42: D986–92. 10.1093/nar/gkt958 24174537 PMC3965079

[30] Jin W, Xu S, Wang H et al. Genome-wide detection of natural selection in African Americans pre- and post-admixture. Genome Res 2012; 22: 519–27. 22128132 10.1101/gr.124784.111 PMC3290787

[31] Mariappan MM, Feliers D, Mummidi S et al. High glucose, high insulin, and their combination rapidly induce laminin-β1 synthesis by regulation of mRNA translation in renal epithelial cells. Diabetes 2007; 56: 476–85. 10.2337/db05-1334 17259394

[32] Rothe J, Thor D, Winkler J et al. Involvement of the adhesion GPCRs latrophilins in the regulation of insulin release. Cell Rep 2019; 26: 1573–84.e5. 10.1016/j.celrep.2019.01.040 30726739

[33] Rorsman P, Ashcroft FM. Pancreatic β-cell electrical activity and insulin secretion: of mice and men. Physiol Rev 2018; 98: 117–214. 10.1152/physrev.00008.2017 29212789 PMC5866358

[34] Sabeti PC, Schaffner SF, Fry B et al. Positive natural selection in the human lineage. Science 2006; 312: 1614–20. 10.1126/science.1124309 16778047

[35] Voight BF, Kudaravalli S, Wen X et al. A map of recent positive selection in the human genome. PLoS Biol 2006; 4: e72. 10.1371/journal.pbio.0040072 16494531 PMC1382018

[36] Sabeti PC, Varilly P, Fry B et al. Genome-wide detection and characterization of positive selection in human populations. Nature 2007; 449: 913–8. 10.1038/nature06250 17943131 PMC2687721

[37] Racimo F, Sankararaman S, Nielsen R et al. Evidence for archaic adaptive introgression in humans. Nat Rev Genet 2015; 16: 359–71. 10.1038/nrg3936 25963373 PMC4478293

[38] Abascal F, Acosta R, Addleman NJ et al. Expanded encyclopaedias of DNA elements in the human and mouse genomes. Nature 2020; 583: 699–710. 32728249 10.1038/s41586-020-2493-4 PMC7410828

[39] Li X, Kim Y, Tsang EK et al. The impact of rare variation on gene expression across tissues. Nature 2017; 550: 239–43. 10.1038/nature24267 29022581 PMC5877409

[40] Zhao X, Emery SB, Myers B et al. Resolving complex structural genomic rearrangements using a randomized approach. Genome Biol 2016; 17: 126. 10.1186/s13059-016-0993-1 27287201 PMC4901421

[41] Redon R, Ishikawa S, Fitch KR et al. Global variation in copy number in the human genome. Nature 2006; 444: 444–54. 10.1038/nature05329 17122850 PMC2669898

[42] Geoffroy V, Herenger Y, Kress A et al. AnnotSV: an integrated tool for structural variations annotation. Bioinformatics 2018; 34: 3572–4. 10.1093/bioinformatics/bty304 29669011

[43] Bae CJ, Douka K, Petraglia MD. On the origin of modern humans: Asian perspectives. Science 2017; 358: eaai9067. 10.1126/science.aai9067 29217544

[44] Fu Q, Li H, Moorjani P et al. Genome sequence of a 45,000-year-old modern human from western Siberia. Nature 2014; 514: 445–9. 10.1038/nature13810 25341783 PMC4753769

[45] Ernst J, Kellis M. Discovery and characterization of chromatin states for systematic annotation of the human genome. Nat Biotechnol 2010; 28: 817–25. 10.1038/nbt.1662 20657582 PMC2919626

[46] Kim DH, Perdomo G, Zhang T et al. FoxO6 integrates insulin signaling with gluconeogenesis in the liver. Diabetes 2011; 60: 2763–74. 10.2337/db11-0548 21940782 PMC3198083

[47] Winkler CA, Nelson GW, Smith MW. Admixture mapping comes of age. Annu Rev Genom Hum Genet 2010; 11: 65–89. 10.1146/annurev-genom-082509-141523 PMC7454031 20594047

[48] Gong H, Pa L, Wang K et al. Prevalence of diabetes and associated factors in the Uyghur and Han population in Xinjiang, China. Int J Environ Res Public Health 2015; 12: 12792–802. 10.3390/ijerph121012792 26473908 PMC4627000

[49] Tao Y, Mao X, Xie Z et al. The prevalence of type 2 diabetes and hypertension in Uygur and Kazak populations. Cardiovasc Toxicol 2008; 8: 155–9. 10.1007/s12012-008-9024-0 18777166

[50] Startek M, Szafranski P, Gambin T et al. Genome-wide analyses of LINE–LINE-mediated nonallelic homologous recombination. Nucleic Acids Res 2015; 43: 2188–98. 10.1093/nar/gku1394 25613453 PMC4344489

[51] Lehrman MA, Schneider WJ, Südhof TC et al. Mutation in LDL receptor: Alu-Alu recombination deletes exons encoding transmembrane and cytoplasmic domains. Science 1985; 227: 140–6. 10.1126/science.3155573 3155573 PMC4449727

[52] Wu N, Ming X, Xiao J et al. TBX6 null variants and a common hypomorphic allele in congenital scoliosis. N Engl J Med 2015; 372: 341–50. 10.1056/NEJMoa1406829 25564734 PMC4326244

[53] Dennis MY, Eichler EE. Human adaptation and evolution by segmental duplication. Curr Opin Genet Dev 2016; 41: 44–52. 10.1016/j.gde.2016.08.001 27584858 PMC5161654

[54] Nuttle X, Giannuzzi G, Duyzend MH et al. Emergence of a *Homo sapiens*-specific gene family and chromosome 16p11.2 CNV susceptibility. Nature 2016; 536: 205–9. 10.1038/nature19075 27487209 PMC4988886

[55] Lu D, Lou H, Yuan K et al. Ancestral origins and genetic history of Tibetan highlanders. Am Hum Genet 2016; 99: 580–94. 10.1016/j.ajhg.2016.07.002 PMC5011065 27569548

[56] Lu J, Lou H, Fu R et al. Assessing genome-wide copy number variation in the Han Chinese population. J Med Genet 2017; 54: 685–92. 10.1136/jmedgenet-2017-104613 28705883

[57] Li H, Durbin R. Fast and accurate long-read alignment with Burrows–Wheeler transform. Bioinformatics 2010; 26: 589–95. 10.1093/bioinformatics/btp698 20080505 PMC2828108

[58] Van der Auwera GA, Carneiro MO, Hartl C et al. From FastQ data to high confidence variant calls: the genome analysis toolkit best practices pipeline. Curr Protoc Bioinformatics 2013; 11: 11.0.1–33. 10.1002/0471250953.bi1110s43 PMC4243306 25431634

[59] Fishilevich S, Nudel R, Rappaport N et al. GeneHancer: genome-wide integration of enhancers and target genes in GeneCards. Database (Oxford) 2017; 2017: bax028. 10.1093/database/bax028 28605766 PMC5467550

[60] Li B, Dewey CN. RSEM: accurate transcript quantification from RNA-Seq data with or without a reference genome. BMC Bioinf 2011; 12: 323. 10.1186/1471-2105-12-323 PMC3163565 21816040

[61] Yang X, Yuan K, Ni X et al. AdmixSim: a forward-time simulator for various complex scenarios of population admixture. Front Genet 2020; 11: 601439. 10.3389/fgene.2020.601439 33343638 PMC7744625

[62] Shabalin AA . Matrix eQTL: ultra fast eQTL analysis via large matrix operations. Bioinformatics 2012; 28: 1353–8. 10.1093/bioinformatics/bts163 22492648 PMC3348564

[63] Kidd JM, Sampas N, Antonacci F et al. Characterization of missing human genome sequences and copy-number polymorphic insertions. Nat Methods 2010; 7: 365–71. 10.1038/nmeth.1451 20440878 PMC2875995

[64] Lawson DJ, Hellenthal G, Myers S et al. Inference of population structure using dense haplotype data. PLoS Genet 2012; 8: e1002453. 10.1371/journal.pgen.1002453 22291602 PMC3266881

