# Supplementary material for: Admixture-driven structural variation diversity and its functional implications
Source: Natl Sci Rev. 2025 Nov 21;13(5):nwaf527. doi: 10.1093/nsr/nwaf527 (PMC12908926; doi:10.1093/nsr/nwaf527)
Supplement: nwaf527_Supplemental_Files [file nwaf527_supplemental_files.zip › SupplData.pdf]

MATERIALS AND METHODS

Sample preparation and sequencing

We collected peripheral-blood samples of 92 Uyghurs from Xinjiang Uyghur Autonomous Region, China, and those of 90 Han Chinese from diverse regions (1, 2). Each individual was a third (or more) generation offspring of a non-consanguineous marriage of members of the same nationality within three generations. All samples were collected with informed consent and approved by the Biomedical Research Ethics Committee of Shanghai Institutes for Biological Sciences (No. ER-SIBS-261408). The personal identifiers of all samples, if any existed, were stripped off before sequencing and analysis. All procedures were in accordance with the ethical standards of the Responsible Committee on Human Experimentation and the Helsinki Declaration of 1975, as revised in 2000.

For both the Uyghurs (n = 92) and Han Chinese samples (n = 90), whole-genome sequencing was performed on Illumina HiSeq X Ten following Illumina-provided protocols with standard library preparation in WuXi NextCODE at Shanghai. All samples were performed whole-genome sequencing to an average depth (~30X) using the Illumina HiSeq XTen platform of 150bp paired-end reads. Among the total 182 samples, 7 samples (1 XJU and 6 HAN) were additionally sequenced as replicates which were used as SV calling quality control in the downstream analysis. In addition, we download the high-coverage (>30X) whole-genome sequencing data of 73 west Eurasian samples from Simons Diversity Project (SGDP), which were paired-end sequenced using Illumina HiSeq2000 (3). All the sequencing reads of XJU, HAN, and the SGDP samples were mapped to the human reference genome (GRCh37) with Burrows-Wheeler Aligner (4). The SNV callings were performed using Genome Analysis Toolkit (GATK) (5).

SV discovery

We applied a total of seven different algorithms/software to call SVs from the bam files (Figure S1). These algorithms utilize the read information such as read depth (RD), pair-end (PE), and split read (SR) to detect variants. Firstly, The individual call-set was generated by CNVnator (6), BreakDancer (7), Pindel (8), Lumpy (9) and BreakSeq2 (10) separately, and the callings with length larger than 10kb from PE and SR methods were filtered out. Then the variants from different algorithms were merged by MetaSV (11). We required the variant less than 10-kb must be supported by at least two algorithms. For deletion and duplication, we also included the large variant (>10kb) if it was solely called by CNVnator, as the RD algorithm is more powerful to capture large SVs (12). For duplications with a maximum copy number over 4 in the sample set, or variants where both deletions and duplications coexist within a population, we classify them as multi-allelic copy number variants. Segmental duplications (if they are not copy number variable among the samples studied) were not considered as SVs in this study. For insertion, we called the variants using PopIns (13) which was developed for detecting non-repetitive non-reference sequences on a population level. The PopIns assembled contigs were then aligned to the reference genome by Lastz (14) and NUCmer (15) based on the reported position to determine whether the contig follows a one-end anchored model or a two-end anchored model. According to the anchored model, we further utilized the number of anchoring read pairs and the anchoring scores from PopIns output to filter the insertion results. We removed any variant (except insertion) with a length larger than 2Mb or smaller than 100bp as

well as the variants overlapping with the centromere, telomere, and the reference gap regions. Furthermore, seven XJU, one HAN, and WEU samples were removed due to excessive variants or outliers in the principal component analysis.

To estimate the precision of our detection pipeline, we called the SVs of a well-characterized sample NA12878 from Illumina HiSeq X short-read sequencing data using our developed pipeline, and compared the results to the five datasets called from long-read sequencing platforms via different algorithms: a. PacBio with SMRT-SV (16), b. PacBio with a combined of tools deposited in Genome in a Bottle (GIAB), c. PacBio with pbsv from GIAB, d. PacBio with Sniffles and e. Nanopore with Sniffles (17) as well as two datasets from short-read sequencing but called with Manta (18) and Delly (19). The precision varies among different SV types with an overall of 0.82 (50% reciprocal overlap threshold) to 0.87 (1-bp overlap threshold; Table S1).

SV map construction and genotyping

For the remaining 85 XJU, 89 HAN, and 67 WEU samples, we merged the intersecting variants across samples into SV regions (Figure S1). For deletions and duplications, we sorted the intersecting variants across samples according to their overlapping proportions, and iteratively merged these variants into copy number variable regions (CNVRs) if the reciprocal overlapping proportion is larger than 50%. Then we genotyped CNVRs using CNVnator. If the genotype is inconsistent with the result from MetaSV call-set, we marked the genotype as missing data. We further applied a series of stringent criteria to obtain a set of high-quality SVs for the downstream analysis. Any CNVR satisfied one of the conditions below was removed: 1) the genotypes in our replicated samples were inconsistent; 2) the genotypes failed to pass the Hardy-Weinberg Equilibrium (HWE) test ($P<0.0001$) in any populations; 3) the average mappability of the region calculated from UCSC Genome Browser was smaller than 0.6; 4) the missing rate of the locus was larger than 0.2. For inversions, we applied a similar way to merge and filter the variant sites and genotyped the variants using DIGTYPER (20). For insertions, the genotype was determined based on PopIns results. We further filtered out the inversions and insertions that failed to pass the HWE or with a missing rate>0.2.

Population structure analysis

Principal component analysis (PCA) was used to uncover the population structure of Uyghur from SV data. We mainly focused on the deletions, of which the callings are more reliable compared with other types of SVs. The Uyghur deletions were merged with five SGDP groups, i.e., West Eurasia, East Asia, South Asia, Siberia, and Central Asia using the same criteria as above. PCA was performed by Eigensoft (21).

Under a four ancestral component model, we inferred the ancestry proportion of Uyghur using a linear regression model,

$$AF_{XJU} = \alpha AF_{CAS} + \beta AF_{WEU} + \gamma AF_{EAS} + \delta AF_{SAS} + \varepsilon$$

where AF_{XJU} is the allele frequency of Uyghur, likewise, AF_{CAS}, AF_{WEU}, AF_{EAS} and AF_{SAS} is the allele frequency of Central Asia Siberian, West European, East Asian, and South Asian respectively. ε is the random error. The coefficient α, β, γ, and δ represents the ancestry contribution of Central Asia Siberian, West European, East Asian and South Asian respectively.

Ancestry sharing and ancestry-biased F_{ST}

We conducted the sharing analysis among Uyghur and the two ancestral populations. Here we randomly sampled 50 individuals from the SGDP data for each of the West and East Eurasian populations as source reference ancestries. We took 30 East Asian and 20 Central Asia Siberian individuals for the East Eurasian population, and 18 South Asian, 32 West European for the West Eurasian population. Such sampling guaranteed that the relative proportion was consistent with the estimated ancestry contributions under our admixture model, and also the identical sample size for each group. We further randomly sampled 50 Uyghur individuals to compare the CNV sharing with the reference ancestries.

Under a two-way admixture model, we calculated the Uyghur expected VAF using the weighted West and East Eurasian VAF with the ancestry proportions of each SV site. We calculated Pearson's correlation to assess the concordance between the expected and the observed VAF.

To measure the deviation of the Uyghur VAF from the expectation under a two-way admixture model, we calculated the ancestry-biased F_{ST} for each SV site, i.e., the F_{ST} between the expected and the observed VAF (22).

Simulation of two-wave admixture

Based on the two-wave admixture model (23), we employed a forward simulation software AdmixSim (24) to simulate the genetic drift of SVs in the admixed population without natural selection. We used the SVs on chromosome 1 in WEU and HAN as the ASPs, and the ancestry proportion was set to 0.512 vs 0.488 (WEU vs HAN). The admixture time of the two waves T_1 and T_2 was set to 250 and 50 generations ago respectively (Figure S7). We assumed 25 years per generation, and the second wave admixture contributed half gene flows from the two ancestral populations with the same ancestry proportion. We used the effective population size of 7,500 for the admixed population. At the end of the simulation, 170 haplotypes of the admixed population were generated, which were further used to calculate the ancestry-biased F_{ST} under a neutral scenario. The simulation was repeated 6 times, and the maximum value of the top 1% simulated ancestry-biased F_{ST} among 6 repeats (0.0224) was used as a threshold to identify the SVs that were significantly deviated from the expected. (Figure S7).

For the *PANK2-AS1* cis-regulated variant analysis, we used the SNVs and SVs at the flanking region of the gene (chr20:3275052-4374912) in WEU and HAN as the ancestral source variants and simulated 170 haplotypes for the admixed population using the model and the parameters above.

Natural selection analysis

We conducted an iHS analysis to search for the SVs that were under natural selection for XJU, HAN, and WEU separately. We applied SHAPEIT2 (25) to perform phasing with both SNVs and SVs. Then we used selscan (26) to calculate Z-scores of iHS for each variant (combing SVs and SNVs) in each population. We considered |Z-score|>2 as the candidate signals. As the signals shared by Uyghur and the ancestral populations may reflect the selection signatures in the ancestral population, we further excluded the XJU candidate SVs that were also in the top 5% iHS of the WEU or HAN. In addition, by using selscan we conducted XP-EHH analysis for

XJU against the ancestral population WEU and HAN, respectively. The site with normalized |Z-score|>2 was considered as the locus under positive selection in XJU.

Archaic-origin SVs

Taking advantage of the dense SNVs from NGS data, we first used ArchaicSeeker2.0 (27) to search for archaic introgressed regions in the XJU genomes. The three archaic genomes (a Denisova genome (28), an Altai Neanderthal genome (29) and a Vindija Neanderthal genome (30)) with deep-sequencing data available were included in the analysis. Each XJU sample at the introgressed region was assigned a state '0, 1 or 2' representing that 0, 1, or 2 copies of DNA sequence were introgressed from archaic hominids. An SV is defined as archaic origin if it locates within the introgressed region and co-segregates with the introgressed segment. We required that the introgressed SV genotype must be highly correlated with the status of archaic introgression across all the XJU samples (Pearson's correlation coefficient r=<-0.8 for deletion and r>=0.8 for insertion, inversion, and duplication). We restricted this analysis for deletions and duplications for the reason that they can be genotyped directly in the archaic genomes. We further required the variant is presented in the archaic genome.

Functional annotation of SVs

The genes were annotated with RefSeq. Residual variation intolerance score (RVIS) was used to annotate the gene intolerance (31). The enrichment analysis was done by permutating the identical number and length of the segments to the real data over the genome. The permutation was repeated 1,000 times. When comparing between the populations, we randomly selected 50 samples within each population to perform the functional impact analysis. The random sampling was repeated 100 times for each population.

We annotated the regulatory elements using ENCODE database (32). For other functional element annotations, five annotation classes were included: 1) literature-curated regulatory regions from ORegAnno (33); 2) topologically associated domain (TAD) boundaries generated in GM12878 from GEO accession GSE63525 (34); 3) the consensus set of transcription factor (TF) binding sites from UCSC genome browser (35, 36); 4) ChrommHMM annotations (promotors, genic enhancers, intergenic enhancers, bivalent enhancers, and polycomb repressed elements) from the Roadmap Epigenomics Project (37); 5) PhyloP scores from UCSC genome browser (38).

The clinical impact was annotated with AnnotSV (39), which classified the SVs into five classes as the American College of Medical Genetics and Genomics (ACMG) proposed: Class 1, benign; Class 2, likely benign; Class 3, variant of unknown significance; Class 4, likely pathogenic; Class 5, pathogenic.

Gene expression analysis

We also collected RNA for 90 of the 92 Uyghur samples and 39 out of 90 Han Chinese samples. RNA was extracted using PAXGene Blood RNA Kit (QIAGEN). Each RNA sample was sequenced by Illumina HiSeq2000 platform with ~30× coverage for 100 bp unstrand paired-end reads. We used Trim Galore (0.4.3) to trim adapters of the raw reads. Next, the trimmed reads were mapped to the human reference genome (hg19) using STAR (40). The average mapping rate was ~92.42% (minimum 83.78%; maximum 93.74%). Then, we quantified and

normalized gene expression using RSEM (41). The expressed gene was defined as FPKM > 0.01. In total, there were 20,032 genes for both populations with 20 or more samples expressed, among which 19,352 autosomal genes were used for downstream gene expression analysis. The eQTLs were mapped with MatrixEQTL (42) with adjusted expression values and SVs under a linear regression model. We considered the cis-variants that are within upstream or downstream 1-Mb of the gene boundaries on the same chromosome to perform the eQTL analysis. The significant eQTLs were identified with a false discovery rate (FDR) of 5% at the gene level. To assess the contribution of cis-SVs to the gene expression compared with the cis-SNVs, we used the framework as ref. (43) with some modifications that we considered the heritability of gene expression with all cis-SVs as the fixed effect and all the cis-SNVs as the random effect. We employed the R package varComp (44) to perform the analysis.

To screen for the ancestral rare/low-frequency SV pairs that jointly regulate gene expression in the same direction, we focused on the variants with VAF<0.05 in both WEU and HAN. Considering SV calling/genotyping accuracy, we restricted the eQTL analysis for the variant pairs that met the two conditions: i) at least one sample in XJU carrying both variant alleles; ii) the homozygous variant-allele carrier could be observed in our XJU samples for both variants. The SNV-SV pair analysis was performed similarly. The framework of eQTL analysis was generally identical to the eQTL analysis of Uyghur specific genetic combination Scenario II, with the modification of one variable as the copy number of the SNVs and the other variable as the copy number of the SVs instead of the ancestry-specific variants (See "Uyghur specific variant combination" section below).

Association of SVs with GWAS SNPs

We used GWAS SNVs as a bridge to link the Uyghur SVs and the reported phenotypic traits in the GWAS catalog (45). We calculated the linkage disequilibrium between each Uyghur SV and GWAS SNVs within 1Mb of the target SV. The SVs showing strong LD ($r^2 \geq 0.8$) with GWAS SNVs were defined as GWAS-associated SVs. For all the GWAS associated SVs in the Uyghur population, we partitioned these variants according to the sharing relationship (i.e., XJU-specific, shared with East Eurasian, shared with West Eurasian, and shared with both Eurasian ancestries) and performed the enrichment analysis of GWAS associated SVs in each sharing group against the rest using Fisher's exact test.

Admixture induced SV diversity

To study the impact of ancestry proportion on the SV diversity, we analyzed *in silico* Uyghur subpopulations with a monotonically increasing ancestry proportion. Under an admixed model of two-way admixture from East and West Eurasians, we first estimated the ancestry proportion for each of the XJU samples. PCAdmix (46) was used to infer the ancestry proportion with combined SNV data, and CHB (Han Chinese in Beijing) and CEU (Utah residents with Northern and Western European ancestry) from the 1000 Genomes project were used as ancestral reference populations (47). Then we ordered the Uyghur samples according to the inferred East Eurasian proportions and sequentially assigned every 40 samples with consecutive ranks of East Eurasian proportions into a subgroup with replacement, i.e., the i-th group contained samples starting with the i-th rank of the East Eurasian proportion and ending with the (i+39)-th rank of the East Eurasian proportion. After the grouping procedure, we have 46 *in*

silico Uyghur subpopulations with monotonically increasing East Eurasian proportions. The number of segregating SVs was calculated for each of the subpopulations. For deletion and duplications, we only included the non-redundant CNVs in the analysis. The correlation measured by R^2 was calculated based on two models (Figure S13). One is a linear regression model as below:

$$y_i = ax_i + b + e$$

where y_i is the number of segregating sites in the i-th population, x_i is the admixture proportion of East Eurasian ancestry in the i-th subpopulation, and e is the random error. We denoted the correlation coefficient of this model as R^2_{line}. In the other model, we added the squared term of the admixture proportion of East Eurasian ancestry,

$$y_i = ax_i^2 + bx_i + c + e$$

where y_i and x_i is the same as above, and the correlation is denoted as R^2_{para}.

To test whether the observed correlation was observed by chance, we performed permutation by shuffling the ancestry proportion for different samples. The two R^2 statistics were calculated as above. The permutation was repeated 1,000 times, and the P-value was calculated as the fraction of results with larger R^2_{para} but with smaller R^2_{line} than the real data, which are 0.887 and 0.512 respectively (Figure S15).

To investigate the relationship between SV and inter-ancestry recombination, we calculated the distance of each SV to the nearest ancestry-switch point. Here the ancestry-switch point was defined as the middle position of the boundaries of two consecutive segments with different ancestries based on the PCAdmix results. Accordingly, the distance was calculated as the start or end position of SV to the ancestry-switch point whichever is smaller, and the corresponding negative and positive sign of the distance indicates whether the SV is on the upstream or downstream of its closest ancestry-switch point, respectively. The distance of non-singleton SVs was the median of the distance across all the SV carriers. To further study the SV mutation mechanism of non-allelic homologous recombination (NAHR), we analyzed the homology within 5kb flanking breakpoints of SVs. The homology here was defined as sequences with length>300 bp and identity>80% by using Lastz. The variants with boundaries flanking by homologous sequences were regarded as NAHR mediated SVs. Only deletion and duplication were considered in this analysis.

Uyghur-specific genetic variant combination

We mainly considered the Uyghur-specific genetic variant combination (USVC) under two scenarios in this study. One is that the two ancestry-specific variants are at the same locus (Scenario I), and the other one is that the two ancestry-specific variants are at different loci but locating within 2Mb on the same chromosome (Scenario II). In both scenarios, we required at least one variant is SV which we refer to as focal SV here. For the former scenario, we focused on one ancestry-specific CNV overlapping with the other ancestry-specific SNV (DEL-SNV or DUP-SNV), and identified 251 ancestry-specific CNVs with at least one ancestry-specific SNVs. After removing the complete linkage SNVs, we obtained 519 USVCs. For the latter scenario, we first considered the types of the two variants were both SVs. We found 2,158 SV-SV pairs satisfying the criteria. Then we considered the following combinations of SVs and SNVs: one ancestry-specific SV with the other ancestry-specific SNV, which resulted in 2,971 SVs with 1,487,788 SV-SNV pairs. The ancestry-specific variant here was defined as meeting

either one of the conditions below: 1) the variant is present in one ancestry (VAF>0.05 in Scenario II) but absent in another; 2) the VAF is ten-fold in one ancestry to another. In this analysis, we also supplemented the SV list with the high-quality variants discovered by long-read sequencing technology in the ASPs (48), if they satisfied the ancestry-specific SV criterion above. We applied FreeBayes (49) to genotype the SNVs in the SV region.

To evaluate the functional impact of these Uyghur-specific configurations, we conducted eQTL analysis for the combinations of the genetic variants and the flanking genes within 2Mb. We applied a linear regression model for the eQTL-analysis as below.

$$Y = \beta_0 + \beta_{EAS}X_{EAS} + \beta_{WEU}X_{WEU} + X\beta + \varepsilon$$

where Y is the gene expression level, X_{EAS} and X_{WEU} are the copies of the East Eurasian and the West Eurasian specific variant (SV or SNV) in the Uyghur population respectively, β_{EAS} and β_{WEU} correspond to the variant effect for the East Eurasian and the West Eurasian specific variant respectively, $X\beta$ is the covariant (age and gender), and ε is a random error drawn from $N(0, \sigma^2 I)$. Multiple testing was performed across all the pairs of variant combinations with the genes in the 2Mb flanking region by using the Benjamini-Hochberg method. To reduce the multiple testing burden, only the SNVs that are not in complete linkage were considered. We excluded the USVCs that could be observed in the ancestral source population, focused on the USVCs that emerged in at least one XJU sample in Scenario I and at least two XJU samples with a homozygous variant in Scenario II. Additionally, we required that the East Eurasian-ancestry allele in XJU had the same direction of effect size towards gene expression as that in the Han Chinese. The results with adjusted P-value<0.1 for both variants were regarded as significant USVCs associated with gene expression.

To infer the local ancestry of the Uyghur-specific variant combinations at the *FOXO6* locus, we applied ChromPainter (50) to the local phased haplotypes in 85 Uyghur samples. WEU and HAN were used as an ancestral reference in the local ancestry inference. Only the regions with 80% consistently inferred ancestry were marked as the West/East Eurasian ancestry, otherwise, the regions were marked as common Eurasian ancestry as their ancestries could not be determined unambiguously.

Evolutionary pattern analysis of Uyghur SVs

By using a sharing approach, we dissected the Uyghur SVs into five classes: a) the ancestral group, representing the variants that emerged before the divergence between archaic hominids and the modern human; b) the ancient group, representing the modern human-specific variants; c) the Out-of-Africa group, denoting the variants that emerged during the modern human out of Africa events; d) the derived group, representing the variants that occurred after the divergence between the west and east Eurasian; e) the Uyghur specific group, denoting the recent and putative post-admixture events. We genotyped all the Uyghur deletions in the SGDP samples and archaic hominids (Denisovan, Vindijia, and Altai Neanderthals). For the ancestral group, we required the Uyghur deletions could be found in both SGDP African samples and any one of the archaic hominids; For the ancient group, we required the Uyghur deletions could be found in SGDP African samples but not in archaic hominids; For the Out-of-Africa group, we required the Uyghur deletions could be found in both SGDP WEU and Han Chinese, but not in the SGDP African group; For the derived group, we required the Uyghur deletions could be found in only one of the ancestries, i.e., either in WEU or Han Chinese, but not in SGDP

African group; For the Uyghur specific group, we required the Uyghur deletions absent in any other groups above.

To identify the putative post-admixture SVs, we used the similar sharing approach but with more stringent criteria: we required that the Uyghur-specific SVs should not overlap with the SVs reported in i) the 1000 Genomes Project Phase III release, 2) SGDP dataset, 3) the gnomAD SV call-set (The overlapping threshold hold was set as 80% reciprocal length). Any SVs sharing with the callings in HAN or the public datasets were regarded as pre-admixture variants. For the comparison analysis, we focused on singleton SVs and control the size between the pre- and post-admixture deletions. We classed all the variants into five-length bins (kb in unit): (0.01, 0.1], (0.1, 1], (1, 10], (10, 100] and (100, 2,000], and randomly sampling 100 deletions with the fraction of the post-admixture variants locating in each bin for each of the pre and post admixture group respectively. The sampling was repeated 1,000 times, and the proportion of eSV, the median effect size, the median PhyloP score, and the fraction of clinical impact were calculated for each sampling.

SUPPLEMENTARY FIGURES

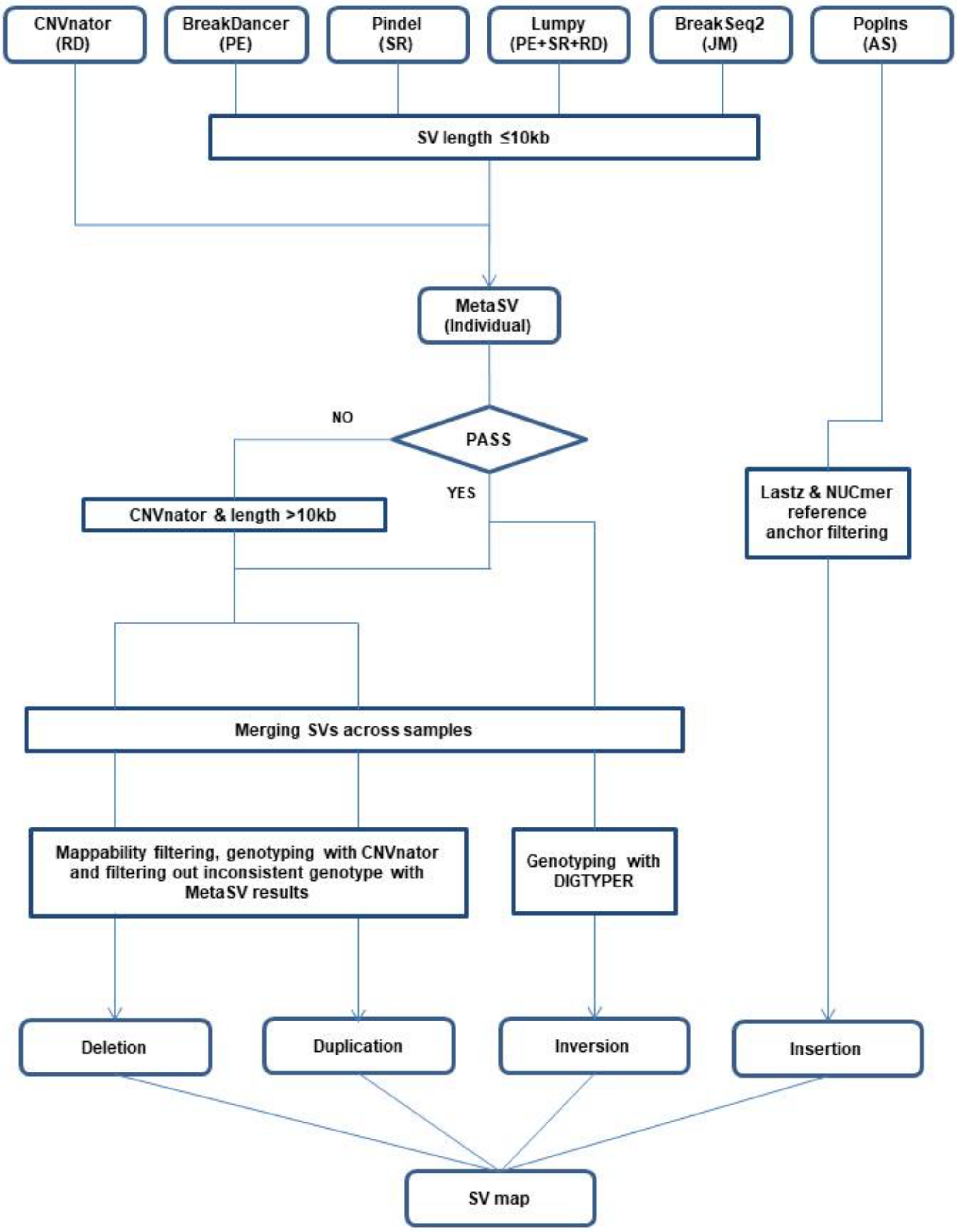

Supplementary Figure 1. SV discovery pipeline.

Briefly, six algorithms (CNVnator, Pindel, BreakDancer, BreakSeq2, Lumpy and PopIns) with different detection methods (read depth, RD; pair end, PE; split read, SR; joint mapping, JM; assemble, AS) were applied to call SVs in each sample. Then the deletions, duplications and inversions were merged and filtered by MetaSV. The insertions were called by PopIns and further filtered with Lastz and NUCmer. Finally the variants were genotyped to construct an SV map (see Methods).

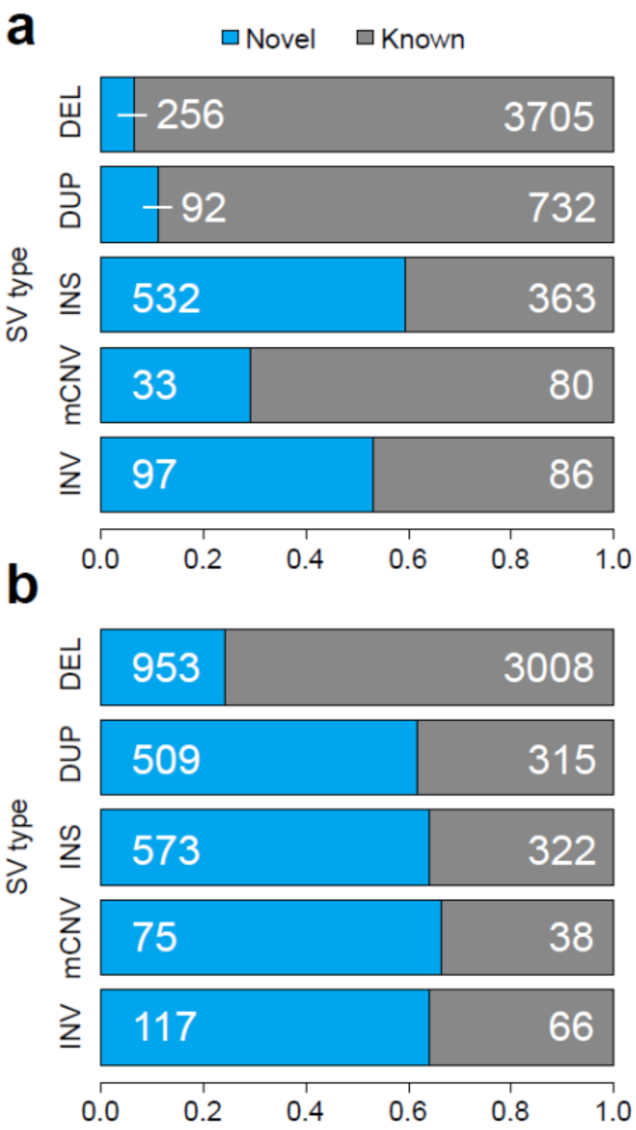

Supplementary Figure 2. Uyghur SVs compared with DGV.

The ‘Known’ represents the XJU SVs have a 1-bp (a) and reciprocal overlapping rate of 50% (b) with the DGV tracks. The ‘Novel’ represents the remaining XJU SVs excluding the ‘Known’. The x-axis shows the proportion of the variants in XJU. An insertion was considered as overlap if it was within 50-bp (a) and 150-bp (b) range of our result and the DGV track.

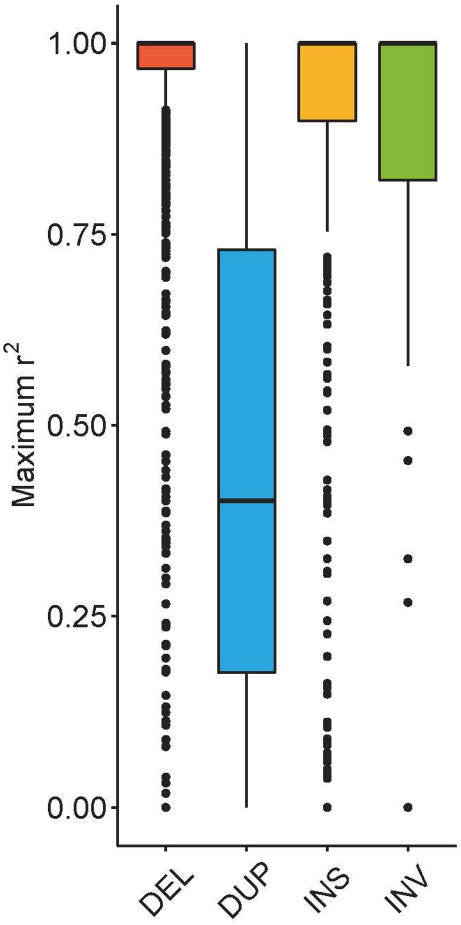

Supplementary Figure 3. Linkage disequilibrium of SVs in the Uyghur population.

The maximum linkage disequilibrium between SV and SNVs in flanking 1Mb region for each SV with minor allele frequency >0.05. Different colours represent different types of SVs. Red, deletion (DEL); blue, duplication (DUP); yellow, insertion (INS); green, inversion (INV).

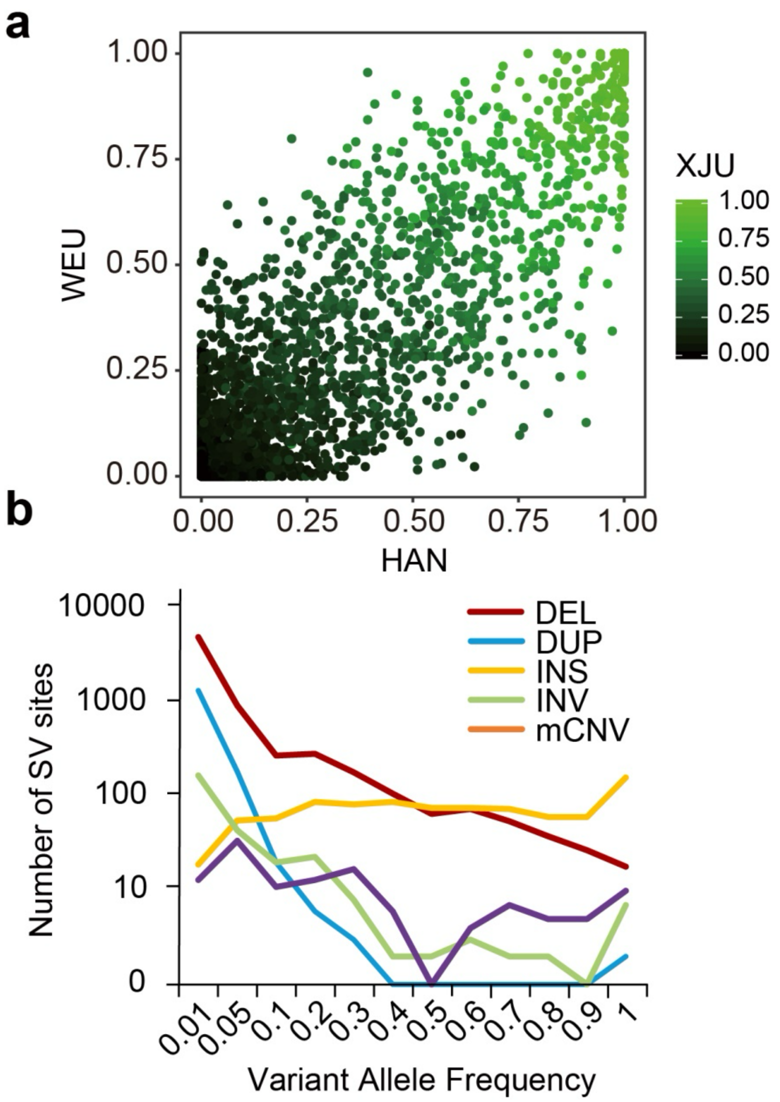

Supplementary Figure 4. Variant allele frequency distribution of SVs in the Uyghur population.

(a) SV allele frequency distribution. Each dot represents an SV. The x-axis, y-axis, and color corresponding to the allele frequency in HAN, WEU, and XJU, respectively. (b) The variant allele frequency (VAF) distribution for all types of SVs in XJU. Red, deletion (DEL); blue, duplication (DUP); yellow, insertion (INS); green, inversion (INV); purple, multi-allelic CNV (mCNV).

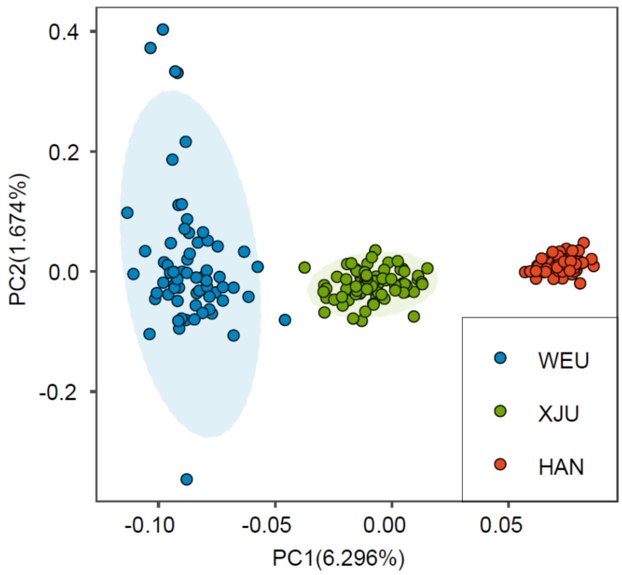

Supplementary Figure 5. Population structure of XJU inferred by PCA using two ancestral reference populations.

The population structure was inferred by using SGDP west Eurasian samples (WEU; blue) and the Han Chinese samples (HAN; red) as ancestral reference populations with 9,839 biallelic SVs. The Xinjiang Uyghur (XJU) population shows a typical admixed pattern in the PCA plot.

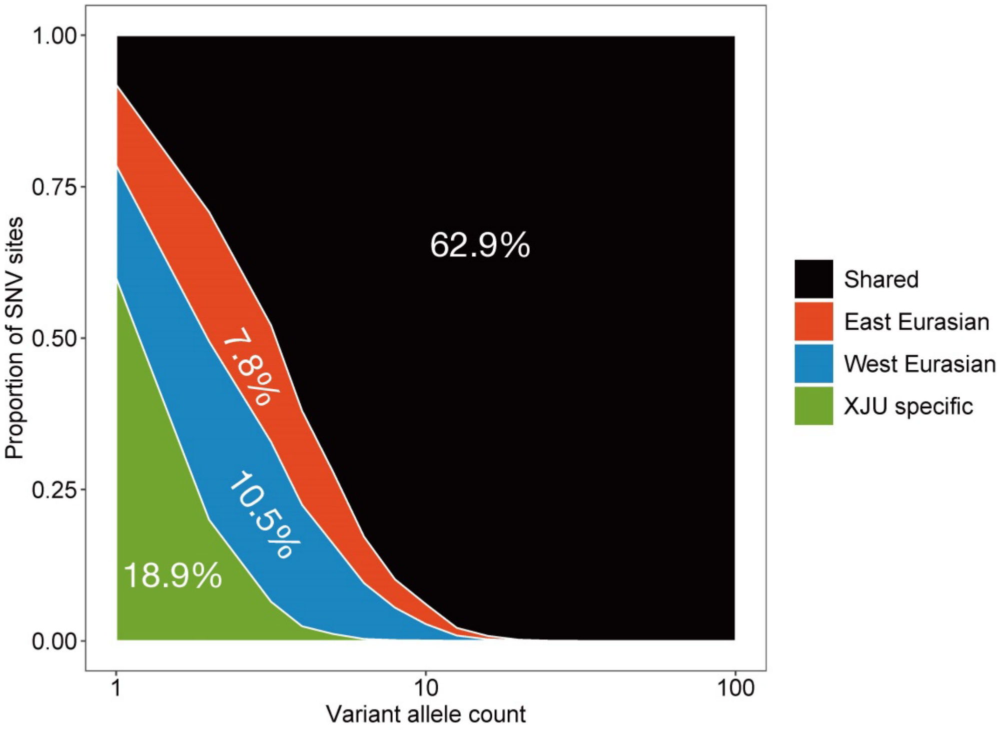

Supplementary Figure 6. SNV sharing between XJU and the ancestral reference populations.

The SNV sharing between 50 XJU samples and 50 samples from each of the west and east Eurasian ancestral reference populations. Only 18.96% of the variants (green) are only observed in XJU. The other variants are shared, of which 62.78% (black) are shared in all populations, and 7.42% (red) and 10.84% (blue) are shared with east and west Eurasians respectively.

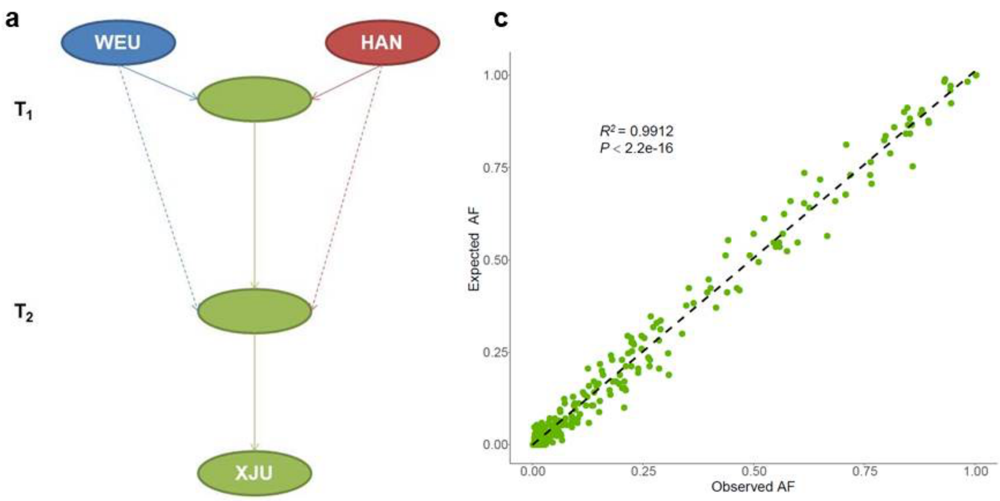

b

Repeat#	1	2	3	4	5	6
Top 1% F_{ST}	0.0213	0.0224	0.0187	0.0158	0.0219	0.0165
Pearson's r^2	0.9906	0.9912	0.9906	0.9914	0.991	0.9922

Supplementary Figure 7. Simulation of two wave admixture.
(a) Two wave admixture model with WEU and HAN as ancestries in the simulation. Assuming 25 years per generation time, the two admixture events occurred in T_1=250 and T_2=50 generations ago, respectively. SVs on chromosome 1 in the HAN and WEU were used as ancestral haplotypes. The effective population size of the admixed population was set to 7,500. At the end of each simulation, 170 haplotypes were generated to match the number of samples in our data. The whole simulation was repeated 6 times. (b) Top 1% value of the ancestry-biased F_{ST} in the simulated data and the Pearson's correlation coefficient of allele frequencies between the simulated and the observed data. (c) The distribution of allele frequencies between the simulated and the observed data on chromosome 1 for the simulation #2. Each green dot represents an SV. The dashed line represents the fitted line using linear regression.

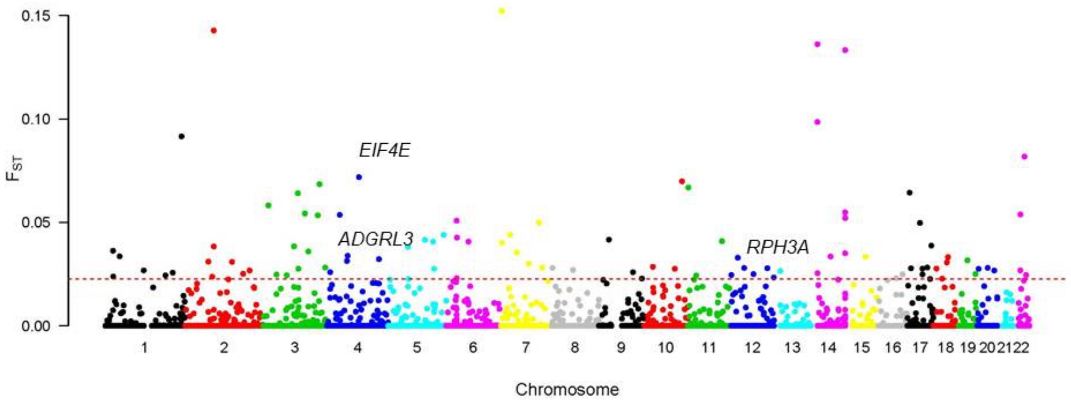

Supplementary Figure 8. Manhatan plot of Ancestry-biased F_{ST} in the Uyghur population. Manhatan plot of ancestry-biased F_{ST} which was calculated between the expected and the observed VAF in Uyghur (Methods). The dashed line represents the threshold of the ancestry-biased F_{ST} with top 1% value (0.0224) in the simulated data.

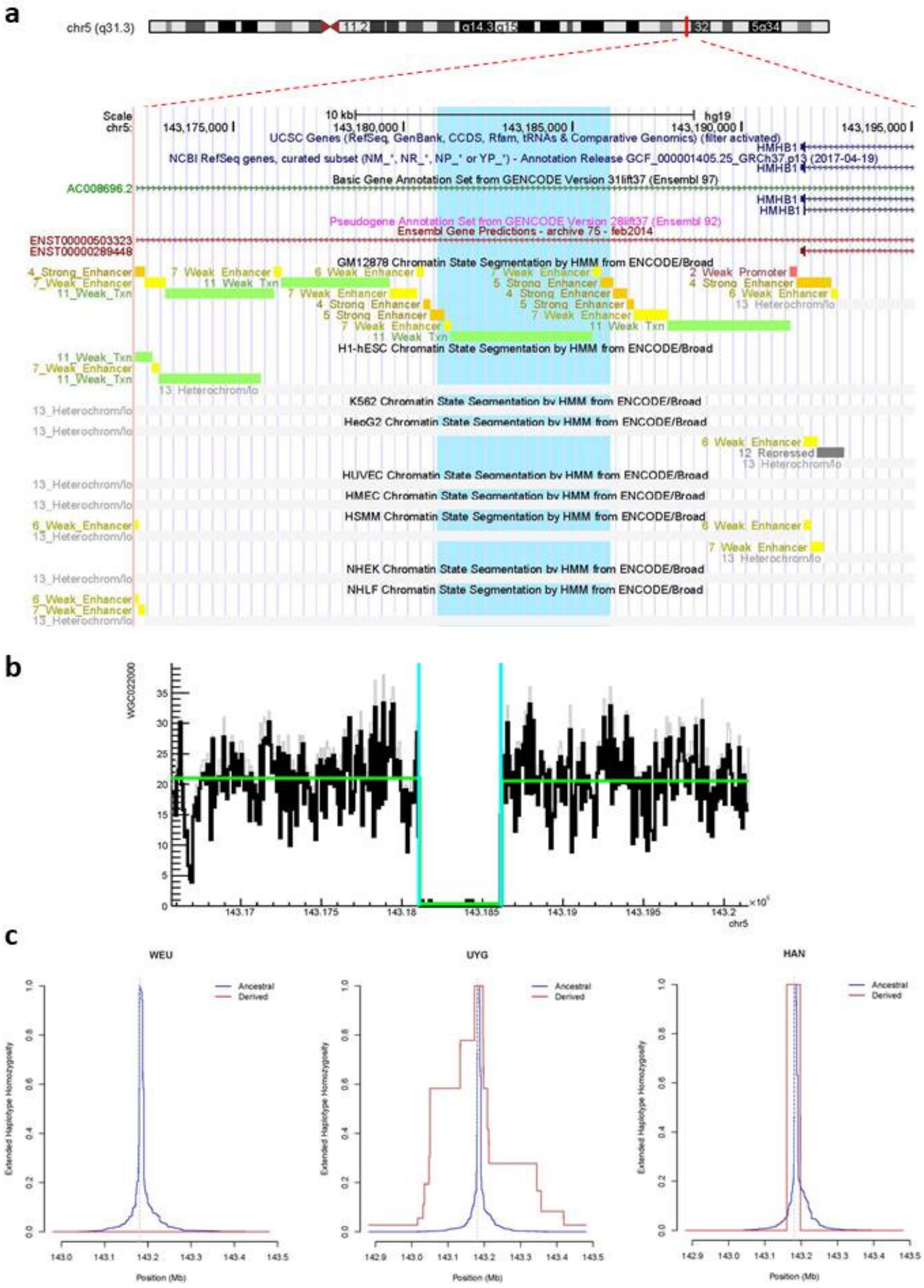

Supplementary Figure 9. Extended haplotype homozygosity of a highly deviated SV in the Uyghur population.

A 5.1kb deletion (chr5:143181043-14318614) among the highly deviated SVs presents longer EHH at the upstream of gene HMHB1. (A) The position of the deletion (light blue region) and the overlapped regulatory elements. (B) Read-depth plot of a homozygous deletion carrier in Uyghur population. Light blue vertical lines indicate the deletion boundaries. (C) EHH plot of the deletion allele (red) versus the normal allele (blue) in three populations.

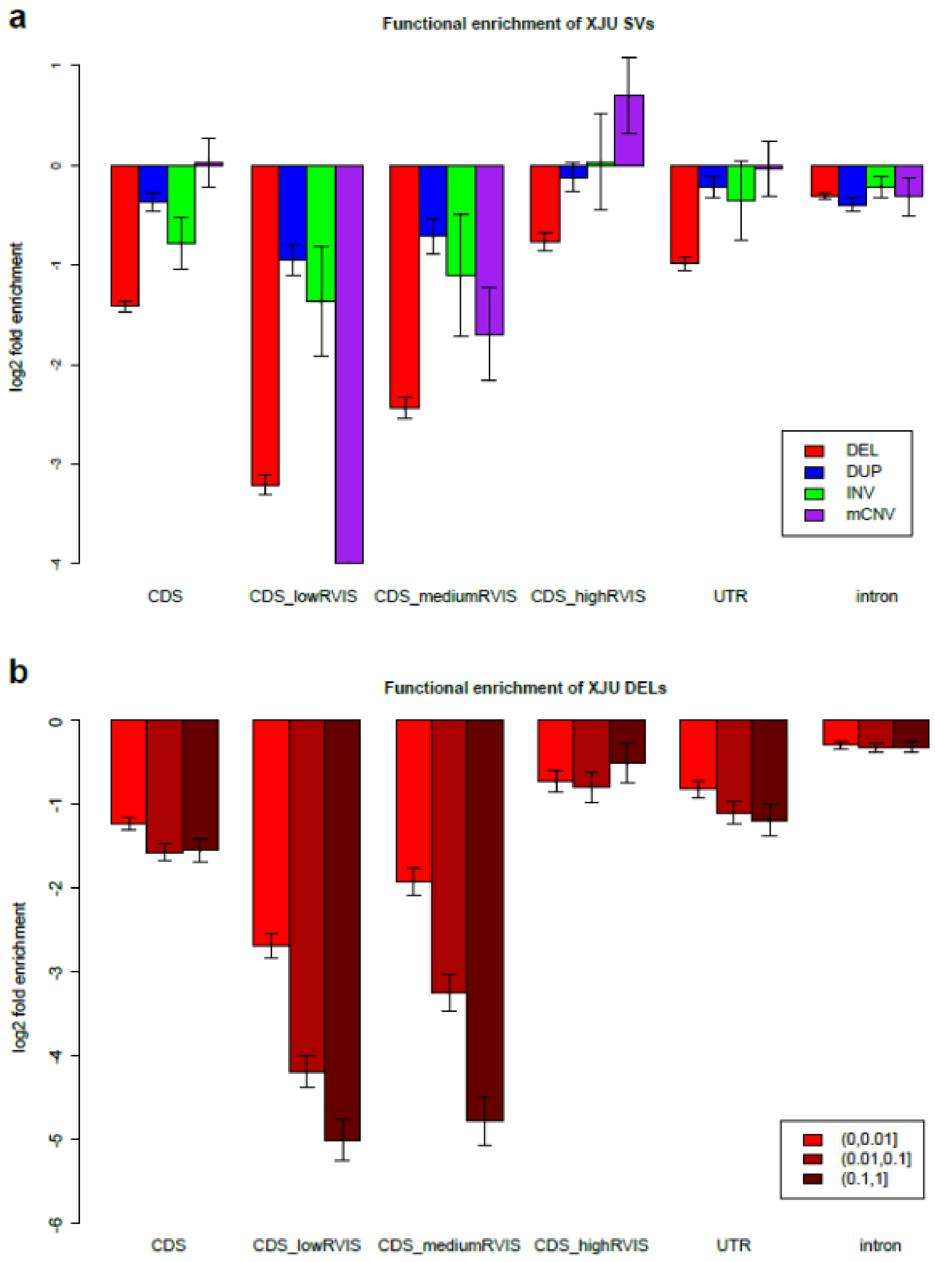

Supplementary Figure 10. Functional enrichment of XJU SVs.

Relative enrichment or depletion of all SV types (a) and deletions with different frequency bins (b) intersecting functional elements compared to a random background model. RVIS: residual variation intolerance score. *no elements intersected. The bars represent the standard deviation of 1,000 permutations.

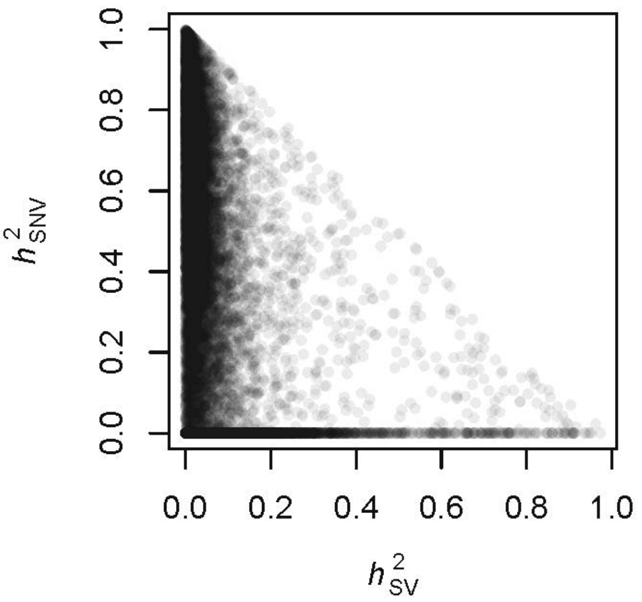

Supplementary Figure 11. Heritability of gene expression partitioned by cis- SVs and SNVs. The relative contribution of all the gene expression for cis-SVs(h_{SV}^2) and cis-SNVs (h_{SNV}^2) was show in x-axis and y-axis respectively. The sum of the contribution from two types of variants for each gene equals to 1.

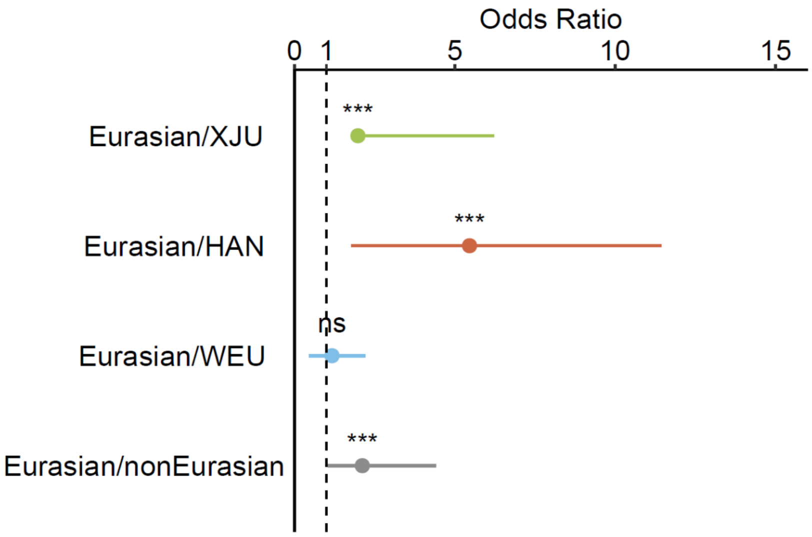

Supplementary Figure 12. Frequency-matched comparison of GWAS-associated SVs in XJU with different ancestry-origin.

The odds ratios were calculated by randomly sampling variants in Eurasian group to match the frequency in the XJU, HAN, WEU and non-Eurasian group. We conducted 1,000 samplings. Dots present the median of 1,000 samplings. Bars represent 95% confidence intervals. 'ns', not significant; ***, P<0.001.

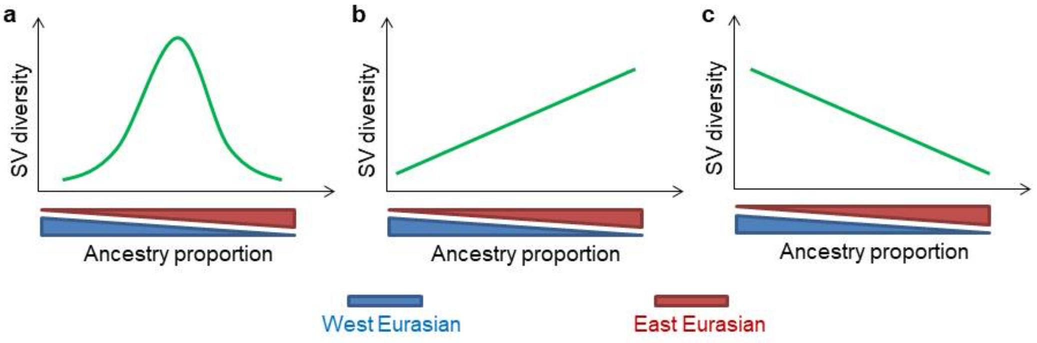

Supplementary Figure 13. Models for admixture-induced SV diversity.

The proposed models for the relationship between the ancestral proportion and the SV diversity in the Uyghur population. (a) The relationship follows a parabola shape, and the maximum diversity occurs when the two ancestry contributes almost equally. (b) and (c) The linear relationship between the ancestral proportion and the SV diversity.

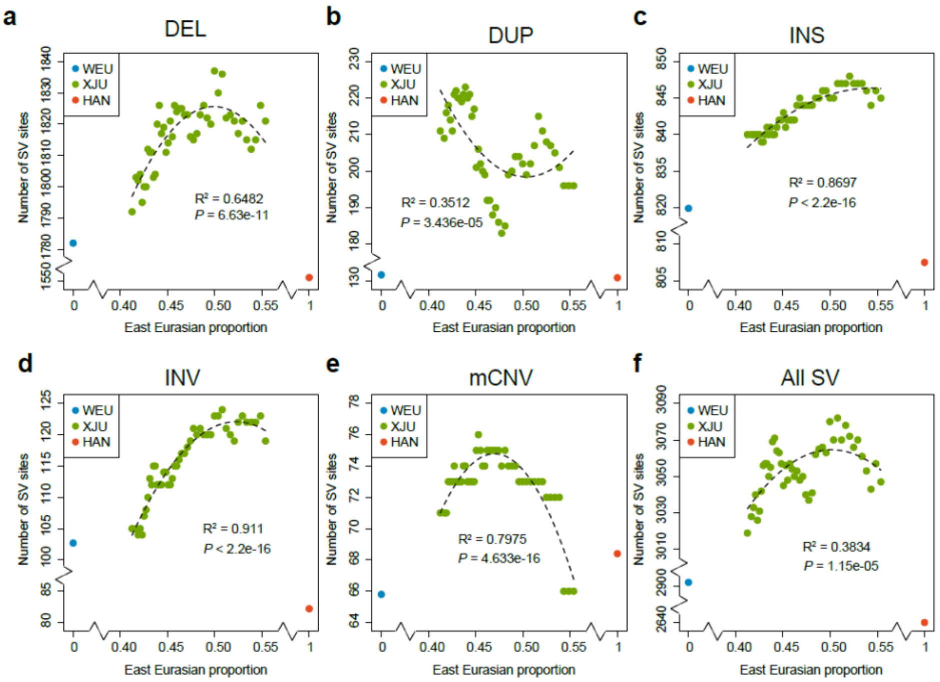

Supplementary Figure 14. Correlation between admixture proportion and the SV diversity in the Uyghur population.

The correlation between diversity and East Eurasian proportion for deletion (a), duplication (b), insertion (c), inversion (d), multi-allelic CNV (e), all SVs (f). Each green dot represents the number of 40 XJU samples (see Methods for details). Blue and red dot represents the mean number of SV sites from 100 samplings with 40 WEU and HAN samples respectively. The dashed lines represent the fitted parabola curve. R^2 and P-values were calculated based on a parabola model.

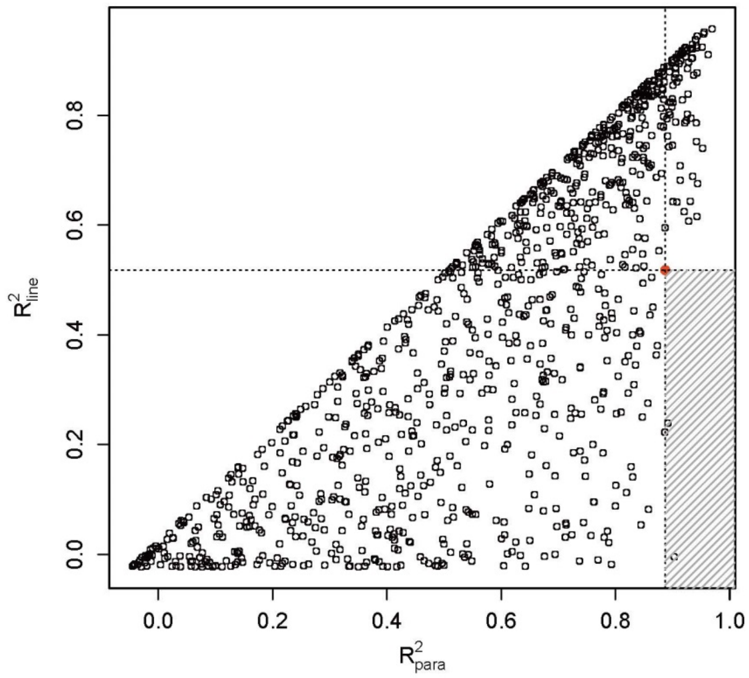

Supplementary Figure 15. Correlation coefficient distribution of the permutation tests for the relationship between ancestry proportion and the genetic diversity.

R^2_{para} and R^2_{line} denote the correlation coefficient of fitting the relationship between ancestry proportion and the genetic diversity using a parabola curve and a linear model respectively. Each dot represents a permutation test. A total of 1000 permutation tests were performed and the red dot showing the observed value in the real data. The shadow area corresponds to the space that has a larger parabola coefficient (R^2_{para} >0.887) and a smaller linear coefficient (R^2_{line} <0.512) than the real data.

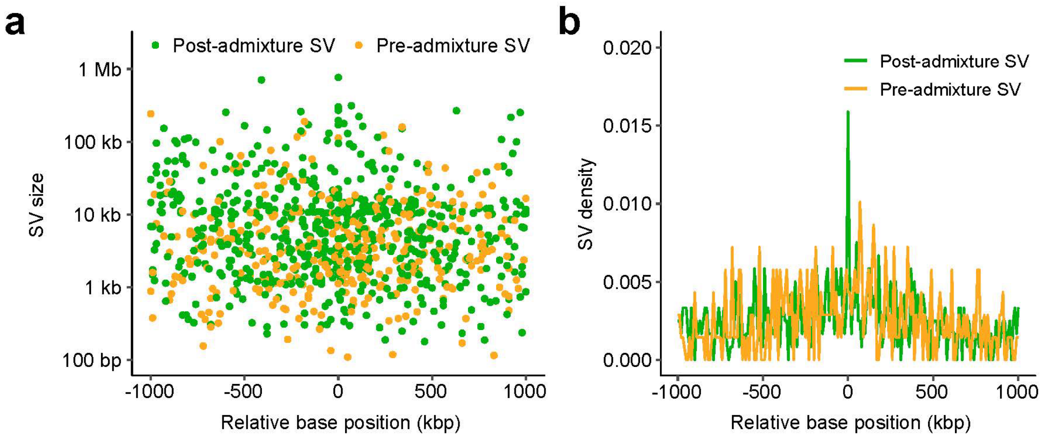

Supplementary Figure 16. Relationship between SVs and inter-ancestry switch points.
(a) Scatter plot of SV length (vertical axis) and the distance to the nearest ancestry-switch point (horizontal axis) for each CNV. There is no significant correlation between the size and the distance for either the pre- or post-admixture variants. (b) Density of SVs with respect to the distance from the nearest ancestry-switch point for the singletons. The pattern is consistent with the results including non-singleton CNVs. Green, post-admixture variants; orange, pre-admixture variants.

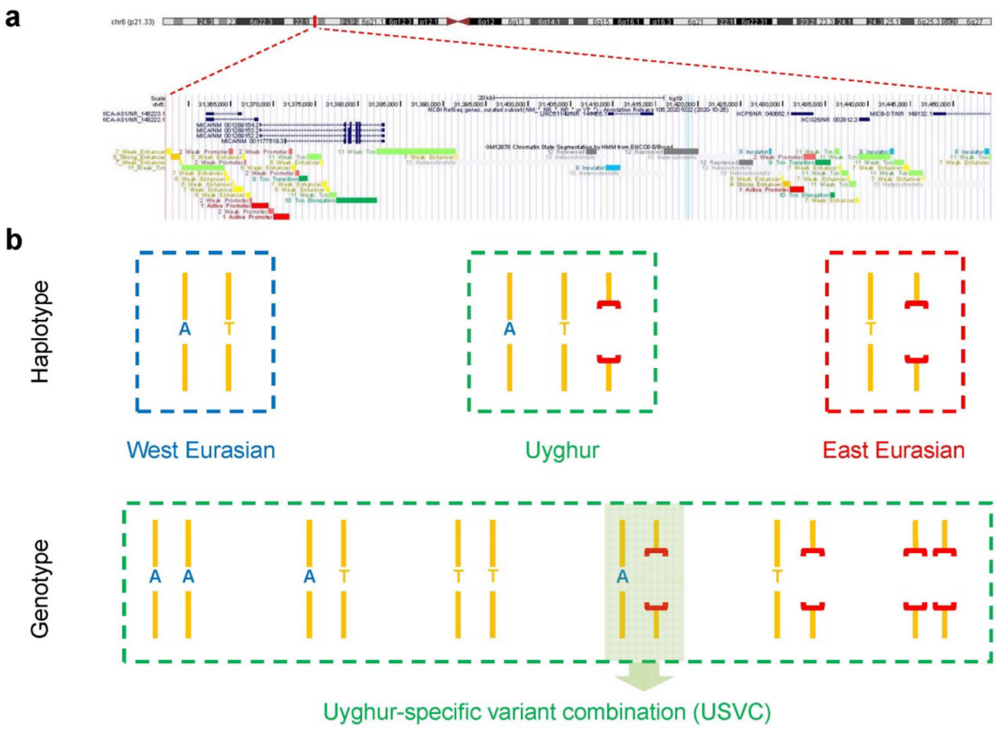

Supplementary Figure 17. Uyghur specific variant combination associated with *HLA-B* expression.

(a) An example of a Uyghur-specific variant combination associated with *HLA-B* expression. The genomic region shows the East Eurasian-specific deletion at chr6:31357451-31454440 and West Eurasian-specific SNV rs3131623 (light blue). Colored boxes show the chromatin state from ENCODE. (b) The haplotype and genotype compositions in the Uyghur population (green box) with alleles from West Eurasians (blue box) and East Eurasians (red box). The shaded green square depicts the Uyghur specific variant combination (USVC).

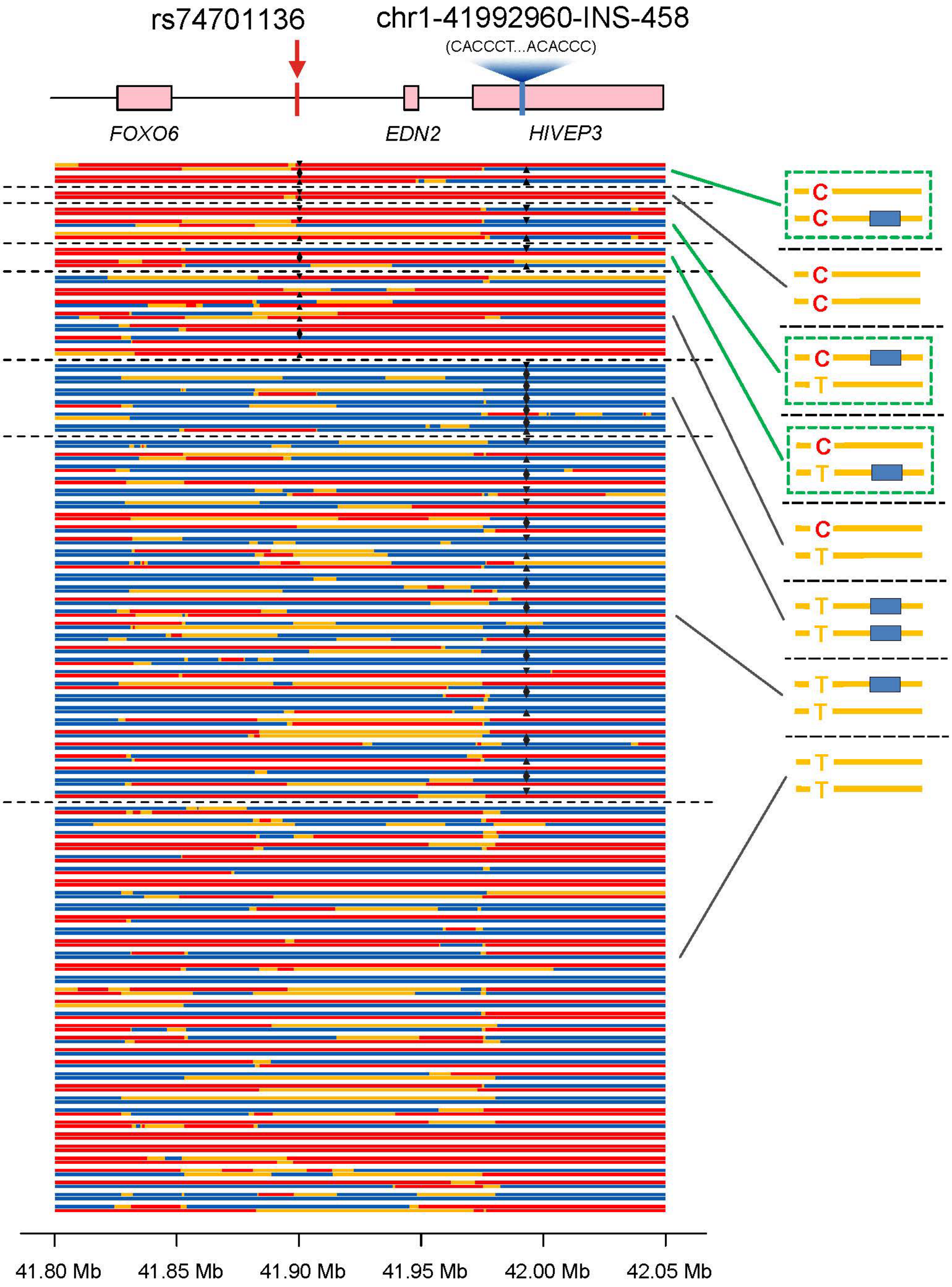

Supplementary Figure 18. Local ancestry inference of haplotypes associated with *FOXO6* expression in 85 Uyghur samples.

Each row on the left panel represents a haploid with inferred ancestry (blue, West Eurasian; red, East Eurasian; Orange, common Eurasian), and every two haploids separated by a blank row represent a diploid genotype in the Uyghur samples. Dashed black lines separate different genotypes. Genes are shown in the pink box at the top. The haplotypes on the right panel are the haplotype diagrams for the two ancestry-specific loci: blue box, insertion allele (chr1-41992960-INS-458; West Eurasian-specific); Orange, reference allele; Red, alternative-allele (East Eurasian-specific). The green dashed boxes indicate the Uyghur specific variant combinations.

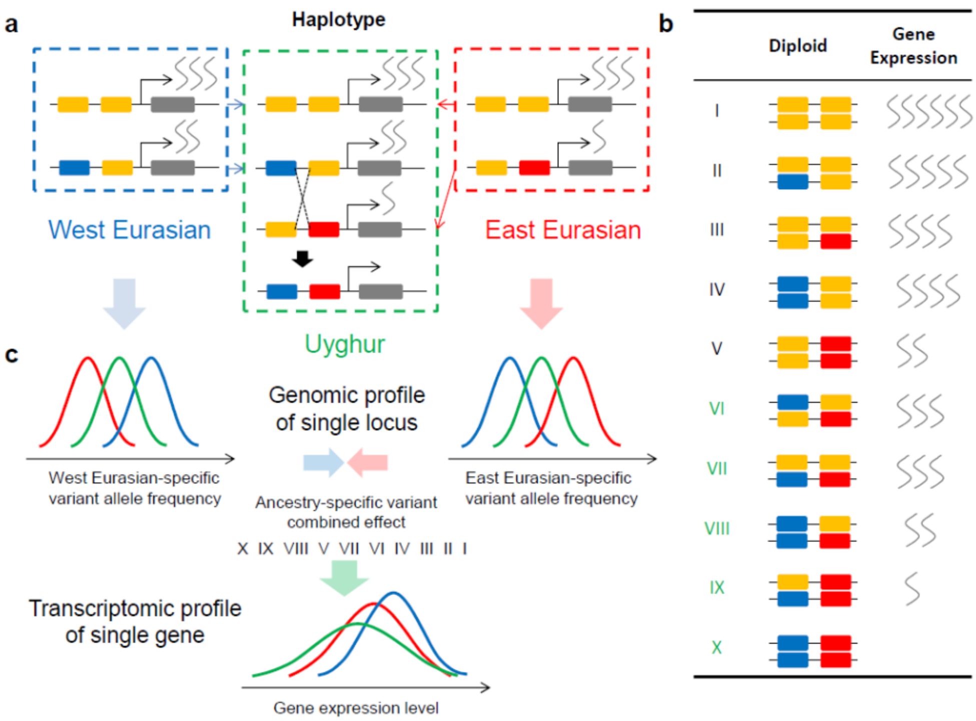

Supplementary Figure 19. Illustration of variant combination effect from genetic diversity to transcriptomic diversity in the Uyghur population.

(a) Haplotype diversity in the West Eurasian (blue box), the East Eurasian (red box) and the Uyghur (green box) population. Each haplotype shows one gene (gray rectangle) on the right side and two genetic variants (orange, reference or wide-type allele; blue, West Eurasian-specific allele; red, East Eurasian-specific allele). The rotated wavy line represents the gene expression levels (mRNA copies). In this toy model, we assumed that: 1) the wide-type haplotype with two reference alleles would produce 3 copies of RNA; 2) the West Eurasian-specific haplotype with one reference and one West Eurasian alleles would produce 2 copies of RNA; 3) the East Eurasian-specific haplotype with one reference and one East Eurasian alleles would produce 1 copy of RNA; 4) The Uyghur-specific haplotype with both ancestry-specific alleles, which is the product of recombination between the West and the East Eurasian haplotype, would yield lowest mRNA. (b) The theoretical diploid genotypes and the associated expression levels in the Uyghur population. The Uyghur-specific diploid genotype was marked in green index. Notably, some Uyghur-specific diploid genotypes (IX, X) would yield even lower expression level compared with the genotypes in the ancestral populations. (c) The upper two diagrams show the density plot of the allele frequency distribution of the West Eurasian specific alleles (left) and the East Eurasian specific alleles (right) in the Uyghur (green) and the two ancestral populations (blue, West Eurasian; red, East Eurasian), respectively. Due to the admixture nature, the expected allele frequency in the admixed population would be in between the two ancestral populations. The lower panel shows the density distribution of gene expression in the Uyghur (green) and the two ancestral populations (blue, West Eurasian; red, East Eurasian). As the combination of the variants would induce a lower expression level than the ancestries, the transcriptomic diversity of the admixed population spans a much wider range

compared with genomic diversity. Here we only showed the toy model that the two ancestry-specific variants both down-regulate the gene expression. Likewise, if the two ancestry-specific variants both up-regulate the gene expression, the combination of the two ancestry specific variants would introduce a higher expression in the admixed population in principle.

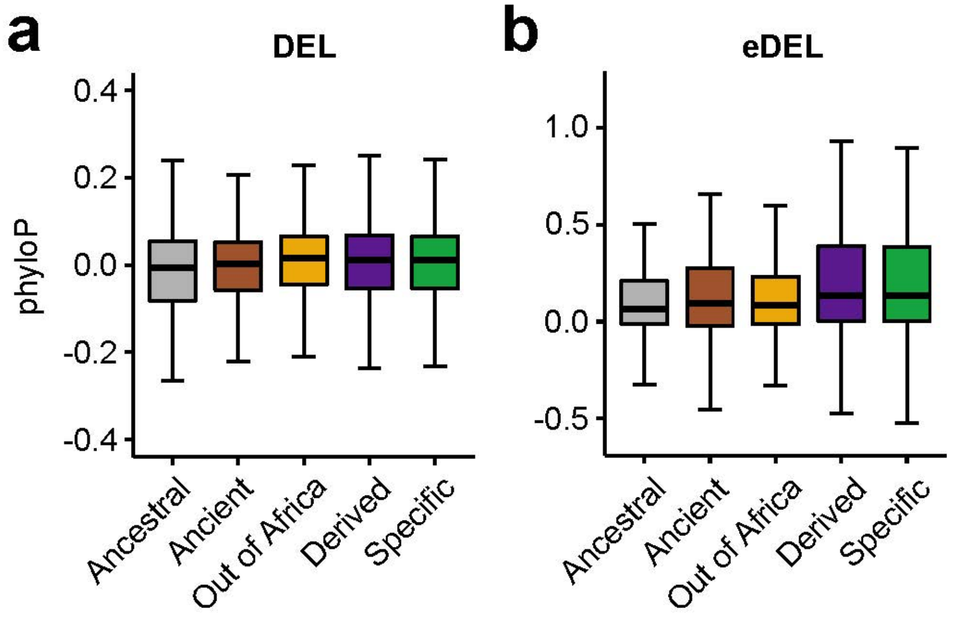

Supplementary Figure 20. PhyloP score among different aged SV classes.

PhyloP score for the deletion (DEL) disrupting genes (a) and eQTL deletion (eDEL) associated genes (b) across different aged variants. Both DEL and eDEL genes show a trend of increasing PhyloP score as the variants become younger ($P<0.005$, Jonckheere's trend test). The thick line represents the median and the bars represent the first and the third quantile.

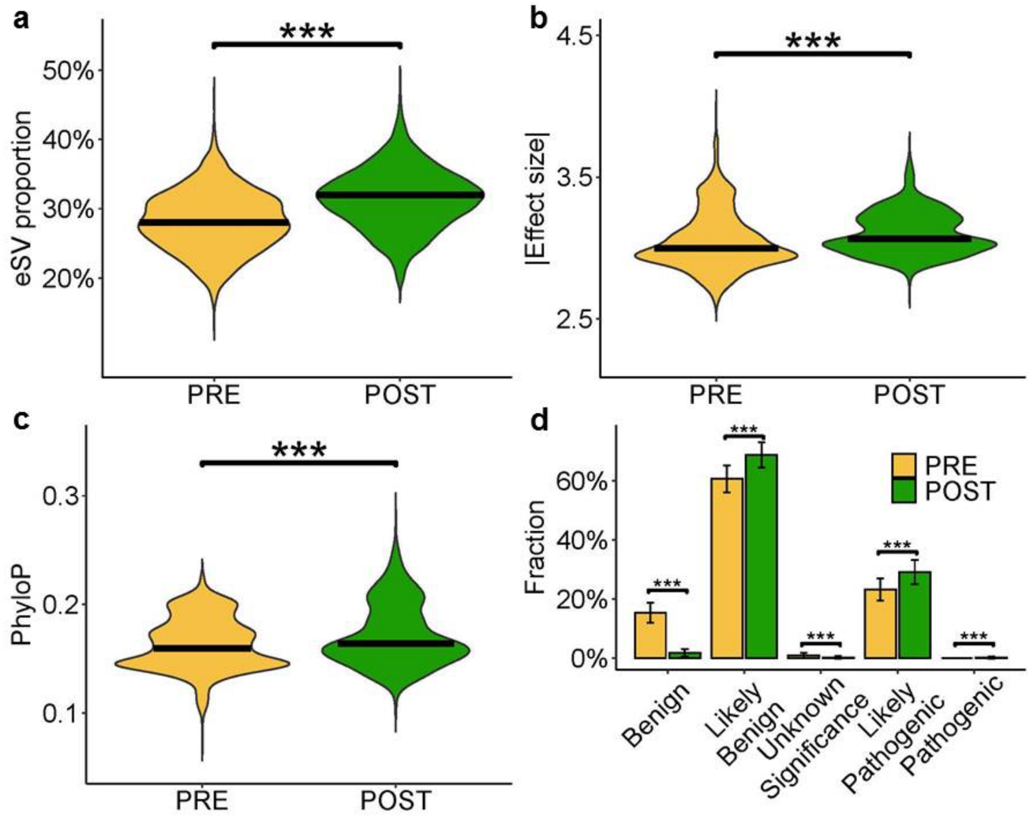

Supplementary Figure 21. Functional impact comparison of size-controlled pre and post-admixture singletons.

eSV proportion (a), absolute value of effect size (b), phyloP score of eGenes (c) and clinical impact (d) comparison of size-controlled pre and post-admixture singleton deletions in 1,000 samplings. For each sampling, 100 singleton deletions were sampled with controlled-size between pre and post-admixture group in (a) and (d); 100 eQTL singleton deletions were sampled with controlled-size between pre and post-admixture group, and the medians in the sampled deletions were recorded for each sampling in (b) and (c). All P-values were based on Wilcoxon test. *, $P<0.05$; **, $P<0.01$; ***, $P<0.001$.

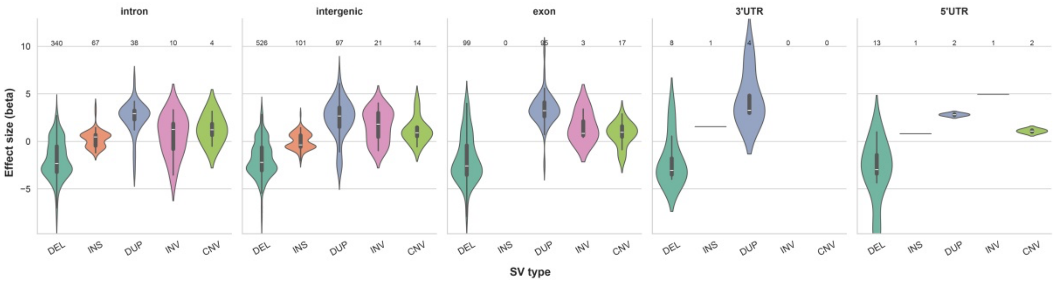

Supplementary Figure 22. Effect size of XJU cis-eSVs in different genomic regions.

Each violin plot shows a distribution of effect size for a type of eQTL SVs located in one kind of genomic regions.

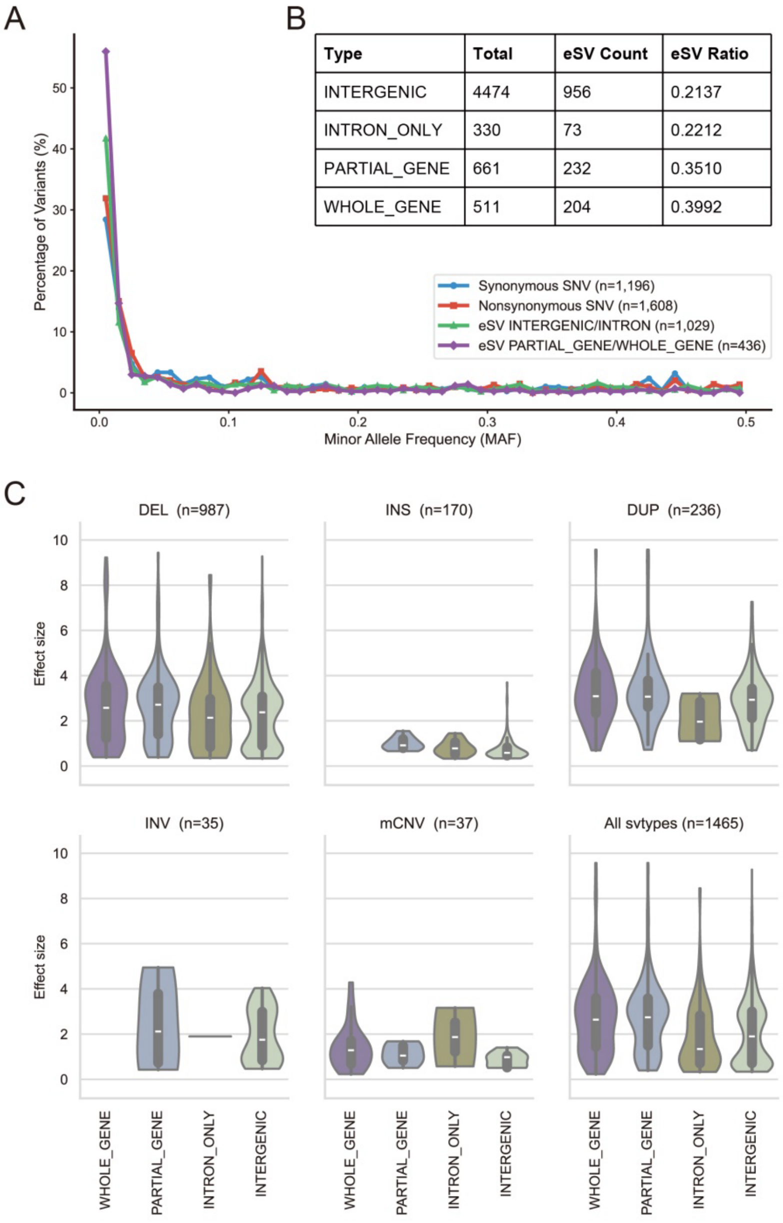

Type	Total	eSV Count	eSV Ratio
INTERGENIC	4474	956	0.2137
INTRON_ONLY	330	73	0.2212
PARTIAL_GENE	661	232	0.3510
WHOLE_GENE	511	204	0.3992

Supplementary Figure 23. Functional importance of coding SVs.

(A) Minor allele frequency spectrum of coding/non-coding SVs and synonymous/non-synonymous SNVs. (B) Proportion of eSV in different SV-gene overlapping categories. (C) Effect size of eSV in different SV-gene overlapping categories in each SV type.

SUPPLEMENTARY TABLES

Supplementary Table 1. Evaluation of SV discovery pipeline. (Separate file, see Table S1.xlsx)

Supplementary Table 2. SV annotations in 67 West Eurasian, 89 Han Chinese and 85Uyghur samples. (Separate file, see Table S2.xlsx)

Supplementary Table 3. Candidate SVs with top iHS and XP-EHH in Uyghur population under natural selection. (Separate file, see Table S3.xlsx)

Supplementary Table 4. Archaic origin SVs in the Uyghur populations. (Separate file, see Table S4.xlsx)

Supplementary Table 5. Significant Uyghur specific variant combinations associated with gene expression. (Separate file, see Table S5.xlsx)

REFERENCES

1. Lu, D, Lou, H, Yuan, K*, et al.* Ancestral Origins and Genetic History of Tibetan Highlanders. *American journal of human genetics*. 2016; **99**(3): 580-94.
2. Lu, J, Lou, H, Fu, R*, et al.* Assessing genome-wide copy number variation in the Han Chinese population. *J Med Genet*. 2017; **54**(10): 685-92.
3. Sudmant, PH, Mallick, S, Nelson, BJ*, et al.* Global diversity, population stratification, and selection of human copy-number variation. *Science*. 2015; **349**(6253): aab3761.
4. Li, H, Durbin, R. Fast and accurate long-read alignment with Burrows-Wheeler transform. *Bioinformatics*. 2010; **26**(5): 589-95.
5. Van der Auwera, GA, Carneiro, MO, Hartl, C*, et al.* From FastQ data to high confidence variant calls: the Genome Analysis Toolkit best practices pipeline. *Curr Protoc Bioinformatics*. 2013; **11**(1110): 11.0.1-.0.33.
6. Abyzov, A, Urban, AE, Snyder, M*, et al.* CNVnator: an approach to discover, genotype, and characterize typical and atypical CNVs from family and population genome sequencing. *Genome Res*. 2011; **21**(6): 974-84.
7. Chen, K, Wallis, JW, McLellan, MD*, et al.* BreakDancer: an algorithm for high-resolution mapping of genomic structural variation. *Nat Methods*. 2009; **6**(9): 677-81.
8. Ye, K, Schulz, MH, Long, Q*, et al.* Pindel: a pattern growth approach to detect break points of large deletions and medium sized insertions from paired-end short reads. *Bioinformatics*. 2009; **25**(21): 2865-71.
9. Layer, RM, Chiang, C, Quinlan, AR*, et al.* LUMPY: a probabilistic framework for structural variant discovery. *Genome Biol*. 2014; **15**(6): R84.
10. Abyzov, A, Li, S, Kim, DR*, et al.* Analysis of deletion breakpoints from 1,092 humans reveals details of mutation mechanisms. *Nature communications*. 2015; **6**: 7256.
11. Mohiyuddin, M, Mu, JC, Li, J*, et al.* MetaSV: an accurate and integrative structural-variant caller for next generation sequencing. *Bioinformatics*. 2015; **31**(16): 2741-4.
12. Kosugi, S, Momozawa, Y, Liu, X*, et al.* Comprehensive evaluation of structural variation detection algorithms for whole genome sequencing. *Genome Biol*. 2019; **20**(1): 117.
13. Kehr, B, Melsted, P, Halldorsson, BV. PopIns: population-scale detection of novel sequence insertions. *Bioinformatics*. 2016; **32**(7): 961-7.
14. Harris, RS. Improved pairwise alignment of genomic DNA. 2007.
15. Kurtz, S, Phillippy, A, Delcher, AL*, et al.* Versatile and open software for comparing large genomes. *Genome Biol*. 2004; **5**(2): R12.
16. Audano, PA, Sulovari, A, Graves-Lindsay, TA*, et al.* Characterizing the Major Structural Variant Alleles of the Human Genome. *Cell*. 2019; **176**(3): 663-75 e19.
17. Ouzhuluobu, He, YX, Lou, HY*, et al.* De novo assembly of a Tibetan genome and identification of novel structural variants associated with high-altitude adaptation. *Natl Sci Rev*. 2020; **7**(2): 391-402.
18. Chen, X, Schulz-Trieglaff, O, Shaw, R*, et al.* Manta: rapid detection of structural variants and indels for germline and cancer sequencing applications. *Bioinformatics*. 2016; **32**(8): 1220-2.
19. Rausch, T, Zichner, T, Schlattl, A*, et al.* DELLY: structural variant discovery by integrated paired-end and split-read analysis. *Bioinformatics*. 2012; **28**(18): I333-I9.
20. Ebler, J, Schonhuth, A, Marschall, T. Genotyping inversions and tandem duplications. *Bioinformatics*. 2017; **33**(24): 4015-23.
21. Patterson, N, Price, AL, Reich, D. Population structure and eigenanalysis. *PLoS Genet*. 2006; **2**(12):

e190.
22. Lou, H, Li, S, Jin, W*, et al.* Copy number variations and genetic admixtures in three Xinjiang ethnic minority groups. *European journal of human genetics : EJHG*. 2015; **23**(4): 536-42.
23. Feng, Q, Lu, Y, Ni, X*, et al.* Genetic History of Xinjiang's Uyghurs Suggests Bronze Age Multiple-Way Contacts in Eurasia. *Mol Biol Evol*. 2017; **34**(10): 2572-82.
24. Yang, X, Yuan, K, Ni, X*, et al.* AdmixSim: A Forward-Time Simulator for Various Complex Scenarios of Population Admixture. *Front Genet*. 2020; **11**: 601439.
25. Yan, LZ, Lin, M, Pan, SY*, et al.* Emerging roles of F-box proteins in cancer drug resistance. *Drug Resist Update*. 2020; **49**.
26. Langefeld, CD, Ainsworth, HC, Graham, DSC*, et al.* Transancestral mapping and genetic load in systemic lupus erythematosus. *Nature communications*. 2017; **8**.
27. Yuan, K, Ni, X, Liu, C*, et al.* Refining models of archaic admixture in Eurasia with ArchaicSeeker 2.0. *Nature communications*. 2021; **12**(1): 6232.
28. Meyer, M, Kircher, M, Gansauge, MT*, et al.* A high-coverage genome sequence from an archaic Denisovan individual. *Science*. 2012; **338**(6104): 222-6.
29. Prufer, K, Racimo, F, Patterson, N*, et al.* The complete genome sequence of a Neanderthal from the Altai Mountains. *Nature*. 2014; **505**(7481): 43-9.
30. Prufer, K, de Filippo, C, Grote, S*, et al.* A high-coverage Neandertal genome from Vindija Cave in Croatia. *Science*. 2017; **358**(6363): 655-8.
31. Petrovski, S, Wang, Q, Heinzen, EL*, et al.* Genic intolerance to functional variation and the interpretation of personal genomes. *PLoS Genet*. 2013; **9**(8): e1003709.
32. Abascal, F, Acosta, R, Addleman, NJ*, et al.* Expanded encyclopaedias of DNA elements in the human and mouse genomes. *Nature*. 2020; **583**(7818): 699-710.
33. Lesurf, R, Cotto, KC, Wang, G*, et al.* ORegAnno 3.0: a community-driven resource for curated regulatory annotation. *Nucleic Acids Res*. 2016; **44**(D1): D126-32.
34. Rao, SS, Huntley, MH, Durand, NC*, et al.* A 3D map of the human genome at kilobase resolution reveals principles of chromatin looping. *Cell*. 2014; **159**(7): 1665-80.
35. Consortium, EP. An integrated encyclopedia of DNA elements in the human genome. *Nature*. 2012; **489**(7414): 57-74.
36. Casper, J, Zweig, AS, Villarreal, C*, et al.* The UCSC Genome Browser database: 2018 update. *Nucleic Acids Res*. 2018; **46**(D1): D762-D9.
37. Roadmap Epigenomics, C, Kundaje, A, Meuleman, W*, et al.* Integrative analysis of 111 reference human epigenomes. *Nature*. 2015; **518**(7539): 317-30.
38. Pollard, KS, Hubisz, MJ, Rosenbloom, KR*, et al.* Detection of nonneutral substitution rates on mammalian phylogenies. *Genome Res*. 2010; **20**(1): 110-21.
39. Geoffroy, V, Herenger, Y, Kress, A*, et al.* AnnotSV: an integrated tool for structural variations annotation. *Bioinformatics*. 2018; **34**(20): 3572-4.
40. Fishilevich, S, Nudel, R, Rappaport, N*, et al.* GeneHancer: genome-wide integration of enhancers and target genes in GeneCards. *Database-Oxford*. 2017.
41. Li, B, Dewey, CN. RSEM: accurate transcript quantification from RNA-Seq data with or without a reference genome. *BMC Bioinformatics*. 2011; **12**: 323.
42. Shabalin, AA. Matrix eQTL: ultra fast eQTL analysis via large matrix operations. *Bioinformatics*. 2012; **28**(10): 1353-8.
43. Chiang, C, Scott, AJ, Davis, JR*, et al.* The impact of structural variation on human gene expression.

Nat Genet. 2017; **49**(5): 692-9.
44. Qu, L, Guennel, T, Marshall, SL. Linear score tests for variance components in linear mixed models and applications to genetic association studies. *Biometrics*. 2013; **69**(4): 883-92.
45. MacArthur, J, Bowler, E, Cerezo, M*, et al.* The new NHGRI-EBI Catalog of published genome-wide association studies (GWAS Catalog). *Nucleic Acids Res*. 2017; **45**(D1): D896-D901.
46. Kidd, JM, Sampas, N, Antonacci, F*, et al.* Characterization of missing human genome sequences and copy-number polymorphic insertions. *Nat Methods*. 2010; **7**(5): 365-U47.
47. Altshuler, DM, Durbin, RM, Abecasis, GR*, et al.* A global reference for human genetic variation. *Nature*. 2015; **526**(7571): 68-+.
48. Ebert, P, Audano, PA, Zhu, Q*, et al.* Haplotype-resolved diverse human genomes and integrated analysis of structural variation. *Science*. 2021.
49. Garrison, E, Marth, G. Haplotype-based variant detection from short-read sequencing2012.
50. Lawson, DJ, Hellenthal, G, Myers, S*, et al.* Inference of population structure using dense haplotype data. *PLoS Genet*. 2012; **8**(1): e1002453.
